# Identification of factors required for m^6^A mRNA methylation in *Arabidopsis* reveals a role for the conserved E3 ubiquitin ligase HAKAI

**DOI:** 10.1111/nph.14586

**Published:** 2017-05-15

**Authors:** Kamil Růžička, Mi Zhang, Ana Campilho, Zsuzsanna Bodi, Muhammad Kashif, Mária Saleh, Dominique Eeckhout, Sedeer El‐Showk, Hongying Li, Silin Zhong, Geert De Jaeger, Nigel P. Mongan, Jan Hejátko, Ykä Helariutta, Rupert G. Fray

**Affiliations:** ^1^ Functional Genomics and Proteomics of PlantsCentral European Institute of Technology and National Centre for Biomolecular ResearchMasaryk University62500BrnoCzech Republic; ^2^ Institute of BiotechnologyUniversity of Helsinki00014HelsinkiFinland; ^3^ Plant Sciences DivisionSchool of BiosciencesUniversity of NottinghamSutton Bonington CampusLoughboroughLE12 5RDUK; ^4^ Research Center in Biodiversity and Genetic ResourcesUniversity of Porto4485‐661 VairãoPortugal; ^5^ Department of Plant Systems BiologyVIB9052GentBelgium; ^6^ Department of Plant Biotechnology and BioinformaticsGhent University9052GentBelgium; ^7^ Key Laboratory of Crop Gene Resources and Germplasm Enhancement on Loess PlateauMinistry of AgricultureTaiyuanShanxi030031China; ^8^ The State Key Laboratory of AgrobiotechnologyThe School of Life SciencesThe Chinese University of Hong KongHong KongChina; ^9^ School of Veterinary Medicine and SciencesUniversity of NottinghamSutton BoningtonLoughboroughLE12 5RDUK; ^10^ Sainsbury LaboratoryUniversity of CambridgeCambridgeCB2 1LRUK

**Keywords:** *Arabidopsis*, HAKAI, mRNA methylation, *N*6‐adenosine methylation (m^6^A), protoxylem, VIRILIZER

## Abstract

- *N*6‐adenosine methylation (m^6^A) of mRNA is an essential process in most eukaryotes, but its role and the status of factors accompanying this modification are still poorly understood.
- Using combined methods of genetics, proteomics and RNA biochemistry, we identified a core set of mRNA m^6^A writer proteins in *Arabidopsis thaliana*.
- The components required for m^6^A in *Arabidopsis* included MTA, MTB, FIP37, VIRILIZER and the E3 ubiquitin ligase HAKAI. Downregulation of these proteins led to reduced relative m^6^A levels and shared pleiotropic phenotypes, which included aberrant vascular formation in the root, indicating that correct m^6^A methylation plays a role in developmental decisions during pattern formation.
- The conservation of these proteins amongst eukaryotes and the demonstration of a role in writing m^6^A for the E3 ubiquitin ligase HAKAI is likely to be of considerable relevance beyond the plant sciences.

## Introduction

More than 150 nucleotide modifications of RNA have been described and of these > 10 have been reported in mRNA (Machnicka *et al*., 2013). *N*6‐methyladenosine (m^6^A) is the most prevalent internal mRNA modification found in eukaryotes, and has received a burst of interest in recent years (Meyer & Jaffrey, 2014; Fray & Simpson, 2015; Yue *et al*., 2015). m^6^A appears to be involved in a broad range of biological processes including mRNA export from the nucleus (Fustin *et al*., 2013), regulation of splicing (Alarcón *et al*., 2015b; Haussmann *et al*., 2016; Lence *et al*., 2016), mRNA translatability and stability (Wang *et al*., 2014a,b, 2015; Bodi *et al*., 2015; Zhou *et al*., 2015), alternative polyadenylation site choice (Ke *et al*., 2015) and other mechanisms accompanying RNA maturation (Meyer & Jaffrey, 2014; Yue *et al*., 2015). m^6^A is essential for the earliest stages of pattern formation in plants (Zhong *et al*., 2008; Bodi *et al*., 2012; Shen *et al*., 2016) and metazoans (Meyer & Jaffrey, 2014; Geula *et al*., 2015; Yue *et al*., 2015; Haussmann *et al*., 2016; Lence *et al*., 2016), linked with diseases in humans and other mammalian species (Jia *et al*., 2011; Zheng *et al*., 2013) and is required for meiosis in *Saccharomyces cerevisiae* (Clancy *et al*., 2002). Reduced levels of m^6^A also affect circadian period (Fustin *et al*., 2013) and are critical for stem cell differentiation in mammals (Geula *et al*., 2015).

Although m^6^A can be present throughout the primary transcript, it is enriched at 3′ ends, particularly within the last exon of mature mRNA (Bodi *et al*., 2012; Dominissini *et al*., 2012; Li *et al*., 2014; Luo *et al*., 2014; Ke *et al*., 2015; Wan *et al*., 2015). m^6^A is found within a conserved consensus sequence (G/A)(G/A) **A**CU (with a preference for (G/A)G**A**CU) in different eukaryotes (Horowitz *et al*., 1984; Narayan & Rottman, 1988; Dominissini *et al*., 2012; Meyer *et al*., 2012; Luo *et al*., 2014; Wan *et al*., 2015). However, only a subset of potential sites is actually modified and the mechanisms underlying the specificity of writing are not yet understood.

The *S*‐adenosyl methionine dependent methyltransferase METTL3 was first purified and characterized by Bokar *et al*. (1997), as a 70‐kDa subunit of a larger protein complex. Whilst METTL3 was part of a 200‐kDa complex, a further 875‐kDa complex component(s) was required for its *in vitro* activity (Bokar *et al*., 1994, 1997). The identity of any of the other proteins that act together with METTL3 remained unknown until it was shown in *Arabidopsis* that FIP37 (FKBP12 INTERACTING PROTEIN 37) was a partner protein of MTA (the homologue of METTL3) (Zhong *et al*., 2008; Shen *et al*., 2016). Following this initial discovery, the homologues of FIP37, *S. cerevisiae* MUM2 (Muddled Meiosis 2), and mammalian WTAP (Wilms tumour 1 associated protein), were shown to interact with METTL3 (MTA) orthologues and to be required for mRNA methylation in their respective model organisms (Agarwala *et al*., 2012; Ping *et al*., 2014; Wang *et al*., 2014b; Liu *et al*., 2015). More recently, another human methylase, METTL14, phylogenetically related to METTL3 (Bujnicki *et al*., 2002), was shown to form a complex with METTL3 and WTAP, and to be required also for m^6^A formation (Ping *et al*., 2014; Wang *et al*., 2014b; Liu *et al*., 2015).

Other factors in addition to METTL3, METTL14 and WTAP are also involved in m^6^A writing. Human KIAA1429 (Schwartz *et al*., 2014), a homologue of *Drosophila melanogaster* Virilizer (Vir) (Niessen *et al*., 2001), associates with METTL3 and is required for m^6^A writer activity in mammals. Vir was first isolated as a factor that, together with the *D. melanogaster* orthologue of WTAP (Fl(2)D) regulated sex determination (Hilfiker *et al*., 1995; Niessen *et al*., 2001; Ortega *et al*., 2003). Despite sharing many common features with the mammalian methylation process, yeast does not have a homologue of KIAA1429, but an additional protein, SLZ1 (Sporulation‐specific Leucine Zipper 1), absent in humans, is in the yeast complex and is necessary for mRNA methylation activity (Agarwala *et al*., 2012).

Until now, no orthologues of METTL14 and KIAA1429 have been shown to be required for m^6^A writing in organisms distinct from mammals (Meyer & Jaffrey, 2014; Fray & Simpson, 2015; Yue *et al*., 2015). In *S. cerevisiae*, a physical association between IME4 (homologue of METTL3) and KAR4 (homologue of METTL14) has been reported (Ito *et al*., 2001). However, yeast KAR4 lacks a characteristic *S*‐adenosyl methionine binding domain and thus it is likely to perform a different role to mammalian METTL14 (Bujnicki *et al*., 2002; Lahav *et al*., 2007).

Here we report the identification of a conserved set of proteins forming the m^6^A writer complex in *Arabidopsis*. They include MTA (orthologue of human METTL3), MTB (METTL14), FIP37 (WTAP) and VIRILIZER and a homologue of human HAKAI. HAKAI was first characterized in humans as a RING domain E3 ubiquitin‐ligase that mediates the post‐translational downregulation of E‐cadherin at the plasma membrane (Fujita *et al*., 2002). It has recently appeared in animal proteomics interaction lists that include other m^6^A writer complex members (Horiuchi *et al*., 2013); however, until now, a role in m^6^A writing has not been proposed. The demonstration here – that this interaction is conserved across kingdoms and that plant HAKAI is functionally required for full mRNA methylation – may indicate that a similar role for mammalian HAKAI should also be considered.

## Materials and Methods

### Plant growth conditions

Seeds were surface‐sterilized and, after 2 d of stratification at 4°C, cultivated under a 16 h : 8 h photoperiod, 22 : 18°C, light : dark, on 0.5 × Murashige & Skoog medium with 1% sucrose, unless indicated otherwise. For anatomical, histological and reporter gene analyses, primary roots of 4–6‐d‐old vertically grown seedlings were used. Inducible transgene expression was controlled by germinating seeds for 6 d (together with appropriate controls) on sterile media containing 5 μM 17‐β‐estradiol (est) (purchased from Sigma) and documented. In adult plants, 20 μM est was sprayed every other day after rosette formation.

### Plant strains

All *Arabidopsis thaliana* (L.) Heynh. lines were in the Columbia (Col‐0) accession. The following mutants and transgenic plants were described previously: *AHP6prom:GFP* (Mähönen *et al*., 2006), *35Sprom:SR34‐RFP* (Lorković *et al*., 2008) and *ABI3prom:MTA* complemented *mta* SALK_074069 allele (Bodi *et al*., 2012). The T‐DNA insertion SALK_018636 (*fip37‐4*) line was obtained from the NASC Stock Centre (Nottingham, UK) and GABI_217A12 (*hakai‐1*) from GABI‐Kat (Bernd Weisshaar, Bielefeld, Germany) (Kleinboelting *et al*., 2012). The construct for the CRISPR mutagenesis of *hakai‐2* was made by Golden Gate cloning (New England Biolabs, Hitchin, UK) using the vectors and methods described previously (Nekrasov *et al*., 2013). It was designed to make the sgRNA GATTACGGTGGTGGGAGTCA, which targets a site in the first coding exon. Following transformation, T1 plants were initially screened for the presence of an *Mly*I resistant *HAKAI* PCR product and putative homozygous disruption lines were further confirmed by sequencing. Lines homozygous for the *hakai‐2* mutation, but lacking the Cas9 T‐DNA were selected from subsequent generations.

### DNA manipulations and transgenic work

The *vir‐1* phenotype rescuing *VIRprom:GFP‐VIR* was derived from a genomic sequence comprising 2155 bp of the *VIR* promoter region which was fused with GFP in pEPA vector (Ruzicka *et al*., 2010), and subcloned into pML‐BART binary vector (Gleave, 1992). *35Sprom:GS‐VIR* was constructed from *VIR* genomic sequence in pDONR221 recombined into pKNGSTAP (Karimi *et al*., 2007) by a standard Gateway procedure (Invitrogen). MTB, FIP37 and HAKAI mCherry Multisite Gateway based C‐terminal fusions were made as previously described (Karimi *et al*., 2007) and according to the manufacturer's instructions (Invitrogen), using 2374, 1823 and 1680 bp of their native promoter regions, respectively.

The β‐estradiol inducible transgene *WOLprom:XVE*>>*VIR* RNAi and *UBQ10prom:XVE*>>*MTB* RNAi constructs were made as described previously (Mähönen *et al*., 2014; Siligato *et al*., 2016), inserting the regions detailed in Table S1 (Supporting Information) (table of primers used) in the sense and antisense orientation into entry clones with restriction enzyme‐mediated cloning. The promoter *UBQ10* was chosen because it directs stable, widespread expression and is resistant to silencing (Geldner *et al*., 2009). The empty RNAi hairpin was used as a negative control.

### Tandem affinity purification

Cloning of transgenes encoding tag fusions under control of the constitutive cauliflower mosaic virus *35S* promoter and transformation of *Arabidopsis* cell suspension cultures were carried out as described previously (Van Leene *et al*., 2007). Two independent tandem affinity purifications of protein complexes were performed using the GS tag (Van Leene *et al*., 2008) followed by the GS purification protocol as described in Van Leene *et al*. (2011). The protocols of proteolysis and peptide isolation, acquisition of mass spectra by a 4800 Proteomics Analyzer (Applied Biosystems, Framingham, MA, USA), and MS‐based protein homology identification based on the TAIR genomic database, have been described previously (Van Leene *et al*., 2010). Putative false positive interactions were subtracted based on prior experience with *c*. 40 TAP experiments on wild‐type (WT) cultures and cultures expressing TAP‐tagged mock proteins GUS, RFP and GFP (Van Leene *et al*., 2010).

### m^6^A analysis

The quantification of relative m^6^A levels was performed as described previously (Zhong *et al*., 2008). Briefly, 20 μg of total RNA was extracted from *Arabidopsis* seedling samples (or from roots of β‐estradiol treated inducible RNAi lines) using the RNAqueous kit (Ambion), the poly(A)^+^ fraction was purified twice using the MicroPoly(A) Purist kit (Ambion) and the quality of the mRNA checked on an RNA 6000 LabChip, with an Agilent Bioanalyzer (Ambion). For each sample, 50 ng of mRNA was digested with 1 μl of Ribonuclease T1 (1000 units μl^−1^; Fermentas, Altrincham, UK) in a final volume of 10 μl (1× polynucleotide kinase buffer) for 1 h at 37°C and the exposed 5′ end of the digested mRNA fragments labelled using T4 polynucleotidekinase (10 units; Fermentas) and 1 μl [γ‐^32^P] ATP (6000 Ci mmol^−1^; Perkin‐Elmer, Waltham, MA, USA). Following ethanol precipitation, labelled RNA was resuspended in 10 μl of 50 mM sodium acetate buffer (pH 5.5) and digested with P1 nuclease (Sigma‐Aldrich) for 1 h at 37°C. 2 μl of each sample was loaded onto cellulose TLC plates (20 × 20 cm; Merck, Hertfordshire, UK) and developed in a solvent system consisting of isobutyric acid: 0.5 M NH_4_OH (5 : 3, v/v), for the first dimension, and isopropanol : HCl : water (70 : 15 : 15, v/v/v), for the second dimension. Spot intensities were determined using a storage phosphorscreen (K‐Screen; Kodak, Rochester, NY, USA) and Bio‐Rad Molecular Imager FX in combination with Quantity One 4.6.3 software (Bio‐Rad).

### Seedling phenotype analysis

For the quantification of seedling phenotypes, the plates with seedlings were photographed and measured with ImageJ software (Schneider *et al*., 2012). Vertical growth index, as a measure of gravitropic response, defined as a ratio between the root tip ordinate and the root length, was determined as described previously (Grabov *et al*., 2005). Approximately 15–20 seedlings were processed for each treatment, and three independent experiments were performed, giving the same statistically significant results (representative experiments are presented).

For statistical analysis, equal variances of datasets were verified by the Levene test, and the Kruskal–Wallis nonparametric test was performed simultaneously with ANOVA. Data were evaluated with NCSS 2007. The data presented are means ± standard errors.

### Histological analysis and microscopy

Fuchsin staining and confocal imaging were performed on the primary roots of 4–5‐d‐old seedlings as described previously (Mähönen *et al*., 2006). The quantitative analysis of protoxylem phenotypes was performed on fuchsin‐stained roots as described (Bishopp *et al*., 2011). Lugol staining for columella starch granules was carried out as described previously (Friml *et al*., 2002). For the confocal laser scanning microscopy, a Zeiss LSM 780 microscope was used. Due to low signal intensity of transgenes inside the root stele, the contrasts and brightness were enhanced to reveal expression and localization patterns, unless stated otherwise. The analysis of *AHP6prom:GFP* on longitudinal optical sections were acquired with the same confocal settings on all lines in the experiment and the (heat map) RainbowRGB lookup table was applied on unprocessed images by ImageJ in order to demonstrate expression changes (Schneider *et al*., 2012).

### Genetic screening and positional cloning

The *vir‐1* mutant was isolated in an EMS mutagenesis screen for altered pattern of *AHP6prom:GFP* expression. The 970‐kb mapping window between marker nga172 and BAC F2O10 on chromosome 3 was established using 100 F_2_ recombinant plants. Using Illumina whole genome sequencing (CD Genomics, Shirley, NY, USA; Schneeberger *et al*., 2009), we identified three mutations within the mapping window in intragenic regions: one in intronic sequence unrelated to known splicing consensus elements, one that caused a synonymous mutation and one in the 5′ splice site of *VIR* intron 5 that led to mis‐splicing of *VIR* transcripts.

### Sequence analysis and multiple sequence alignment

The domain composition of VIR was examined using Smart (Letunic *et al*., 2012) and Pfam (Punta *et al*., 2012) databases. For creating the multiple sequence alignments, the protein sequences were aligned using the Clustal Omega algorithm (Sievers *et al*., 2011) and graphically visualized by JalView v.2.8.0b1 using default ClustalX colour code (Waterhouse *et al*., 2009).

### RNA sequencing

Three biological replicates each of 5‐d‐old *vir‐1* and the complemented mutant seedlings were harvested directly into RNA stabilizing reagent RNAlater (Ambion). Approximately 1‐mm root tips were excized and used as the tissue source for RNA sequencing. Total RNA was extracted with RNAqueous kit (Ambion) and treated with DNAse (Fermentas, Thermo Fisher Scientific Fermentas, Vilnius, Lithuania). 1–3 μg of total‐RNA was used for isolation of poly(A)^+^ RNA (Dynabeads mRNA purification kit; Ambion). The poly(A)^+^ RNA was reverse‐transcribed to cDNA (SuperScript Double‐Stranded cDNA Synthesis Kit, Invitrogen Life Technologies, Carlsbad, CA, USA). Random hexamers (New England BioLabs) were used for priming the first strand synthesis reaction and SPRI beads (Agencourt AMPure XP, Beckman Coulter, Brea, CA, USA) for purification of cDNA. Illumina compatible Nextera Technology (Illumina, San Diego, CA, USA) was used for preparation of RNA‐seq libraries, employing DNA fragmentation and tagging by *in vitro* cut‐and‐paste transposition; 60 ng of cDNA was used instead of DNA. After the tagmentation reaction, the fragmented cDNA was purified with SPRI beads. In order to add the Illumina‐specific bridge PCR compatible sites and enrich the library, limited‐cycle PCR (five cycles) was performed according to the instructions for the Nextera system with minor modifications. For bar‐coded libraries, 50 X Nextera Adaptor 2 was replaced with a bar‐coded Illumina‐compatible Adaptors from the Nextera Bar Codes kit (Illumina) in PCR setup. SPRI beads were used for purification of the PCR products and the library QC was evaluated by Agilent Bioanalyzer (Agilent, Santa Clara, CA, USA).

Each transcriptome was loaded to occupy 1/4 of the lane capacity in a flow cell. C‐Bot (TruSeq PE Cluster Kit v3, Illumina) was used for cluster generation and Illumina HiSeq2000 platform (TruSeq SBS Kit v3 reagent kit) for paired‐end sequencing. Each biological replicate was sequenced once, producing 100 bp paired‐end reads that were then quality trimmed to a length of 93 bp. The sequencing resulted in *c*. 113 M and *c*. 123 M reads for the complemented *vir‐1* line and *vir‐1*, respectively. The sequencing was done in collaboration with the Finnish Institute for Molecular Medicine, Helsinki, Finland.

### Transcriptome analysis

For testing the effects of *vir‐1* on global splicing with rMats (v.3.2.5; Shen *et al*., 2014), the obtained quality filtered reads in Fastq format were trimmed to 80 nucleotides using the rMats trimFastq Python script. The trimmed reads were then aligned to the TAIR10 genome build using Star (Dobin *et al*., 2013). The WT and *vir‐1* data were compared using the –c parameter set to 0.0001 (0.01% splicing difference). Summary outputs filtered by Fdr (*Q *<* *0.05) from rMats are provided (Table S2).

For analyses based on differential expression, sequences were aligned against the TAIR10 genome using TopHat (v.2.0.8b) with the options –bowtie1 and –no‐discordant in two runs and a mean inner distance between mate pairs of 112 to 133 (SD = 50). The junctions predicted by the first run supplied to the second run. Transcript counts were calculated with HTSeq with features marked in the Ensembl v.72 TAIR10 Gene Transfer Format annotation. Differential gene expression was assessed with the DEseq package (Anders & Huber, 2010). The full procedure is described in (Edgren *et al*., 2011). The GO analysis of complete gene list expressed in the *vir‐1* root tip was done using the AgriGO tools (Du *et al*., 2010) with FDR‐corrected *P‐*value (*Q* value) as a ranking criterion and Hypergeometric test (Hochberg FDR) with 0.05 significance cut‐off. The list of vascular regulators (Caño‐Delgado *et al*., 2010) has been used as a benchmark for defining appropriate GO terms for generating manually edited and updated list of genes required for vascular formation.

### Quantitative RT‐PCR

Quantitative reverse transcription polymerase chain reaction (qRT‐PCR) was carried out as described previously (Furuta *et al*., 2014) using a LightCycler 480 (Roche) with LightCycler 480 SYBR Green master mix (Roche) and the manufacturer's qRT‐PCR program recommendations. Four technical repeats were carried out to assess the gene expression levels. Gene expression was normalized to *UBQ10*, as described previously (Furuta *et al*., 2014).

### Yeast two‐hybrid system

The protein–protein interactions were tested as described previously (Zhong *et al*., 2008; Pekárová *et al*., 2011). Plasmids were constructed by Gateway‐based technology using destination vectors pDEST22 (activation domain) and pDEST32 (binding domain). VIR sequence was split into two parts, corresponding to amino acid residues 1 to 2883 (part 1) and 2584 to 6417 (part 2), referring to the AT3G05680.1 gene model. The interactions were tested in three technical and three biological replicates on media lacking histidine and supplemented with inhibitor of histidine synthesis, 3‐amino‐1,2,4‐triazole (3‐AT; Sigma). The yeast growth was recorded after 4 d.

### Accession numbers

Sequence data for genes described in this article can be found in the Arabidopsis Genome Initiative or GenBank/EMBL databases under the following accession numbers: *AHP6* (At1g80100), *MTA* (At4g10760), *MTB* (At4g09980), *FIP37* (At3g54170), *VIR* (At3g05680) and *HAKAI* (At5g01160). The raw sequences for *vir‐1* transcriptome and rMats outputs have been deposited in the Gene Expression Omnibus (GEO) database, accession GSE97174.

## Results

### Identification of a viable mutant orthologous to the splicing regulator/m^6^A writer protein VIRILIZER/KIAA1429

**Figure 1:**
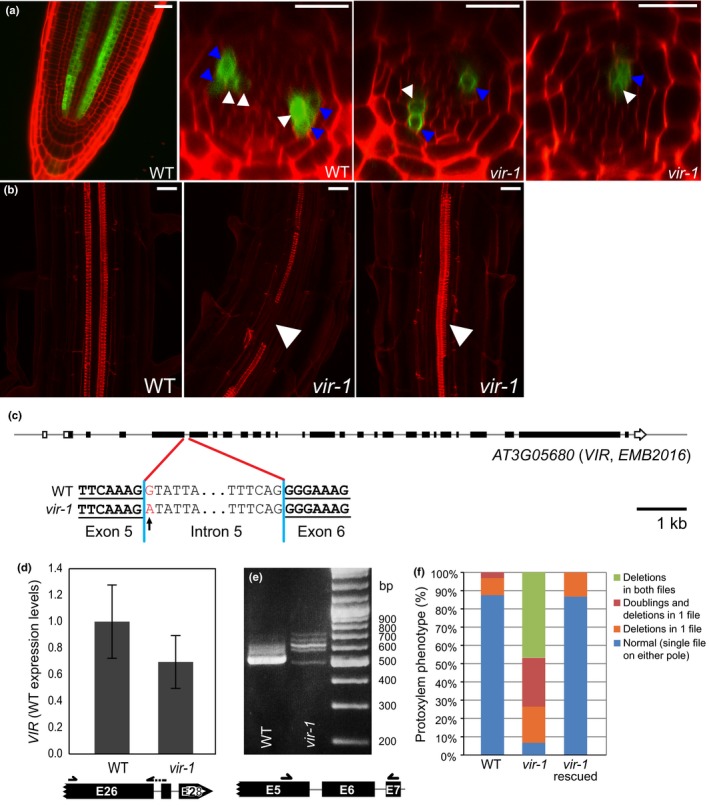
A hypomorphic mutation in the *VIRILIZER* (*VIR*) locus of *Arabidopsis thaliana* leads to vascular defects. (a) Wild‐type (WT) expression pattern of the *AHP6prom:GFP* marker in the root protoxylem founder cells and its aberrant expression in *vir‐1*, as documented by optical longitudinal and cross‐section images. In the WT, the reporter is expressed in one or two files on each side inside the vascular cylinder (giving rise to protoxylem, white arrowheads) and in two accompanying cells of pericycle (blue arrowheads). (b) Fuchsin staining illustrating *vir‐1* protoxylem defects, such as interruptions or doubling of protoxylem (arrowheads). (c) The *vir‐1* mutation is caused by a single nucleotide substitution in the 5′ splice site of the 5th intron of the *VIR* gene. Although the *vir‐1* mutation does not significantly change *VIR* expression levels at α_0.05_ (d), it affects its correct splicing as assayed by reverse transcription polymerase chain reaction (RT‐PCR) around mutated site in *vir‐1* (e). (f) Quantification of the *vir‐1* protoxylem phenotype, which can be rescued by introducing a *GFP‐VIR* transgene into *vir‐1* plants. Quantitative RT‐PCR (d) data are represented as means ± SE. Bars, 20 μm.

The histidine pseudophosphotransmitter AHP6 is a factor required for protoxylem formation in *Arabidopsis* and is the earliest marker of root protoxylem development (Mähönen *et al*., 2006). In a mutant screen designed to identify regulators of *Arabidopsis* vascular development, we isolated one line that exhibited reduced and irregular *AHP6prom:GFP* expression (Fig. 1a) accompanied by defects in protoxylem development in the primary root (Fig. 1b). We used positional cloning and whole genome sequencing to identify *EMB2016* (Tzafrir *et al*., 2004) as the disrupted gene (Fig. 1c). This gene of unknown function was described previously as essential (Tzafrir *et al*., 2004) and is homologous to the *D. melanogaster* sex determination splicing factor, Virilizer (Hilfiker *et al*., 1995; Niessen *et al*., 2001), and to human KIAA1429, associated with m^6^A formation in mammals (Schwartz *et al*., 2014; Fig. S1). Based on the homology to the prototypical gene, we refer to this mutant (and gene) as *virilizer‐1* (*vir‐1*). The *vir‐1* EMS‐induced mutation results in a G‐to‐A conversion at the first nucleotide of intron 5, leading to numerous predominantly wrongly spliced *VIR* transcripts with minor but detectable content of the correct *VIR* mRNA (Figs 1c–e, S2a). The *vir‐1* mutant exhibited pleiotropic phenotypes, which included aberrant root cap formation, gravity response and lateral root development, as well as defective cotyledonal development (Fig. S2b–f). Because these aberrant phenotypes were rescued by complementation with a *VIR* (Figs 1f, S2g), and phenocopied by inducible *VIR* RNAi lines (Fig. S2h), we concluded that we had isolated a viable hypomorphic *virilizer* allele.

### Transcriptome of *vir‐1* mutant

*Drosophila melanogaster* Vir is required for promoting certain alternative splicing events associated with female gametogenesis and X chromosome dosage compensation (Hilfiker *et al*., 1995; Ortega *et al*., 2003; Haussmann *et al*., 2016; Lence *et al*., 2016). We isolated poly(A)^+^ RNA from *vir‐1* root tips and carried out Illumina RNA sequencing. We examined the changes in alternative splicing in *vir‐1* using rMats tools (Shen *et al*., 2014). Only 22 retained introns, one mutually exclusive exon, and eight skipped exon, one alternative 5′ and 13 alternative 3′ splice sites (FDR < 0.01) were identified (Table S2). Given the selected threshold (see the Materials and Methods section), we conclude that the *vir‐1* mutation does not result in extensive alternative or mis‐splicing of transcripts.

Gene ontology (GO) analysis (Du *et al*., 2010) based on the differentially expressed genes in the *vir‐1* mutant (Table S3) revealed that the *vir‐1* mutation affects a range of processes which include those associated with response to environmental cues, metabolic processes and macromolecular localization, and also growth and development (Table S4). The root tip tissue‐specific context allowed us to examine expression of early vascular genes (Table S5). Out of 138 vascular formation related genes identified in our dataset, 35 have been misexpressed, which suggests that vascular development in *vir‐1* is affected at multiple levels (Table S6). Altogether, the observed changes in the *vir‐1* root tip transcriptional profiles suggests that VIR is likely involved in regulation of gene expression, but the function of VIR is rather general than specific and knock‐down of *VIR* does not affect overall splicing rates in *Arabidopsis*.

### VIR closely associates with a conserved set of proteins linked with m^6^A writing

**Figure 2:**
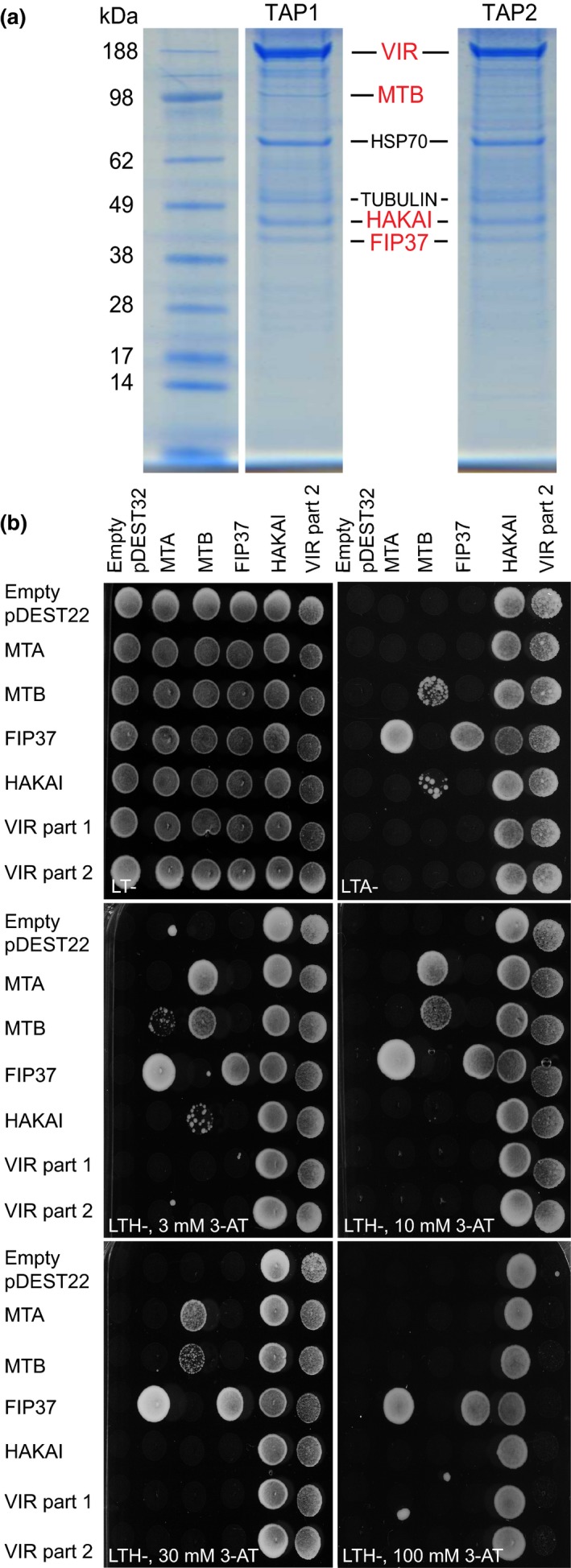
VIRILIZER (VIR) associates with m^6^A writer proteins in *Arabidopsis thaliana*. (a) In tandem affinity purification (TAP), VIR (bait) co‐purifies with FIP37 and HAKAI and in replicate 1 also with the putative *N6*‐adenosyl methylase MTB; TAP1 and TAP2 are two independent TAP experiments; HSP70 and TUBULIN were considered as nonspecific binding proteins, as described in the Materials and Methods section. (b) In the yeast two‐hybrid assay, putative writer proteins fused to activation (pDEST22 vector) and binding (pDEST32 vector) domains show pairwise interactions under various stringency conditions – as documented by growth on selective media lacking with leucine, tryptophan and histidine (‐LTH) and supplemented with various concentrations of 3‐AT to balance basal expression of the *HIS3* reporter, or lacking leucine, tryptophan, and adenine (‐LTA).

Although it is a relatively large protein (236 kDa), VIR lacks well‐characterized protein domains and its precise molecular function remains obscure. The mammalian homologue of VIR was recently shown to associate with m^6^A writer proteins (Ortega *et al*., 2003; Horiuchi *et al*., 2013; Schwartz *et al*., 2014), we therefore decided to test experimentally whether a similar association also existed in *Arabidopsis*. We used tandem affinity purification (TAP) followed by proteolysis and mass spectrometry (Van Leene *et al*., 2011) to identify proteins that associated with the VIR‐GS bait in *Arabidopsis* suspension cell cultures. Using this approach, we consistently identified FIP37 (Zhong *et al*., 2008) and HAKAI (Fig. S3; Fujita *et al*., 2002; Horiuchi *et al*., 2013) co‐purifying with VIR and MTB (Bujnicki *et al*., 2002) also significantly enriched with a lower confidence score (Fig. 2a; Table S7).

In order to further characterize these putative members of the m^6^A writer complex and to examine their relationship to the known plant m^6^A methylase MTA, we tested several pairwise interactions between them using the yeast two‐hybrid system (Y2H) (Fig. 2b). In addition to the known MTA‐FIP37 interaction (Zhong *et al*., 2008), we observed heterodimerization of the *Arabidopsis* MTA and MTB, consistent with the reported interaction between their mammalian orthologues METTL3 and METTL14 (Liu *et al*., 2014; Ping *et al*., 2014; Wang *et al*., 2014b). We also found that MTB but not MTA formed homodimers in Y2H. These data support the findings of Liu *et al*. (2014) who reported that mammalian methylases METTL3 and METTL14 can form heterotetramers. In addition, FIP37 also gave self‐interaction and a consistent though weaker interaction was also seen between HAKAI and MTB. Due to lower interaction fidelities of very large proteins in Y2H (Koegl & Uetz, 2007), we split VIR protein into two parts and also used it for Y2H. However, no interaction was seen with the N‐terminal fragment and only strong transactivation or no interaction was seen with the carboxy terminal fragment in our test system (Fig. 2b). In summary, we show that, in addition to MTA and FIP37 (Zhong *et al*., 2008), MTB, VIR and HAKAI are also associated in the *Arabidopsis* m^6^A writer complex.

**Figure 3:**
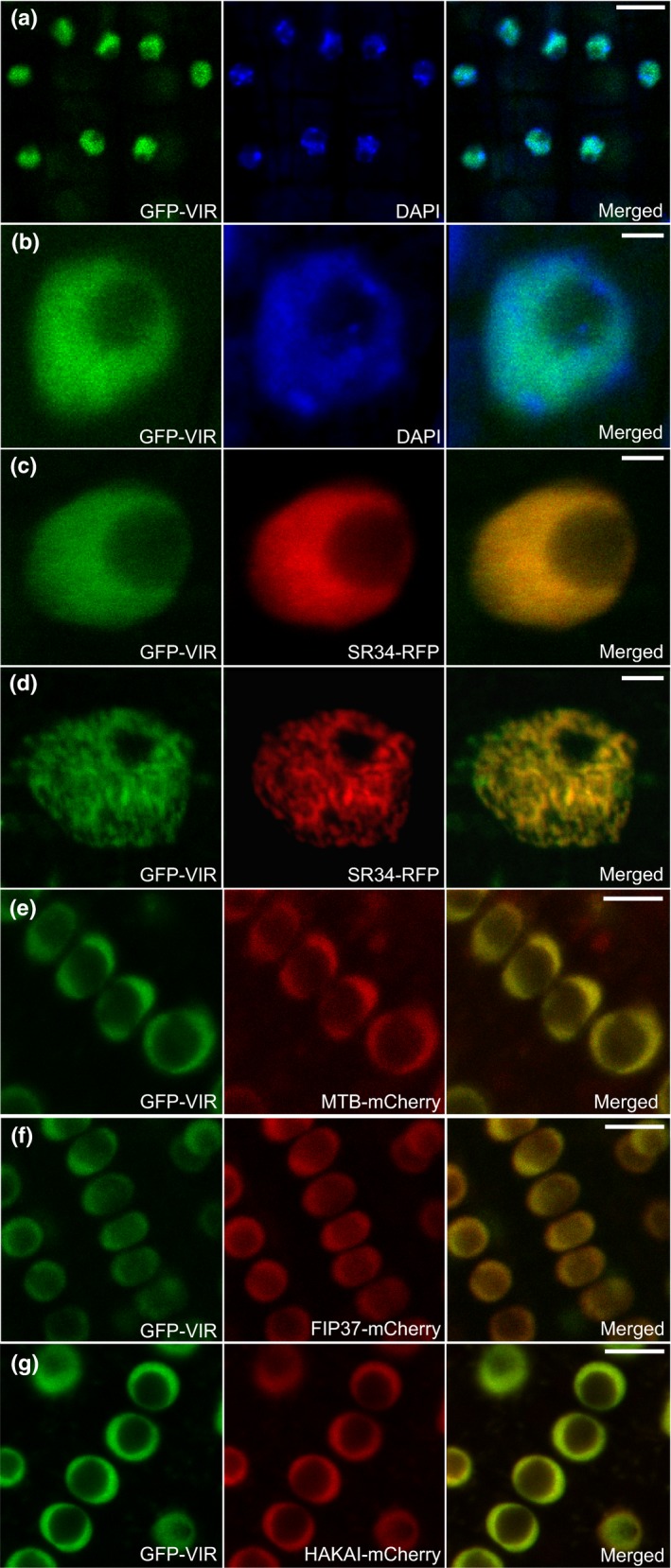
*Arabidopsis thaliana* proteins linked with m^6^A writer activity are nuclear localized. (a, b) GFP‐VIR co‐localizes with fluorescent DNA‐binding dye DAPI. (c, d) GFP‐VIR co‐localizes with nuclear localized splicing factor SR34‐RFP. Smooth nuclear fluorescence distribution was seen in the root tip (c), the characteristic punctate subnuclear compartments (splicing speckles) were observed in the differentiated cells above the root elongation zone (d). (e–g) GFP‐VIR co‐localizes with MTB (e), FIP37 (f) and HAKAI (g) fused to mCherry in root‐tip nucleoplasm. Bars: (a, e–g) 10 μm; (b–d) 2 μm.

Because this conserved set of associating proteins co‐purify, one would expect that they would be found together in the same cell types. To address this question with a complementary approach, we used confocal microscopy to examine the localization of each protein fused with mCherry fluorescent tag, stably transformed into a line expressing a functional *VIRprom:GFP‐VIR* construct (Fig. 1f). In each case, the native promoters were used to drive the expression of the coding sequences of the fusion proteins. We first characterized the GFP‐VIR fluorescence. The signal was detected in the nucleoplasm of all root tip cell types (Figs 3, S4). There was no clear fluorescence indicative of enriched localization of GFP‐VIR in nucleoli, the cytoplasm or in foci resembling (pro)plastid organelles (Fig. 3). In the root meristem nuclei, the GFP‐VIR showed an even signal distribution (Fig. 3c), whilst the differentiated cells above the root elongation zone exhibited a punctate nuclear distribution pattern (Fig. 3d), similar to known splicing factors (Fang *et al*., 2004; Tillemans *et al*., 2006). In order to test the idea that this punctate pattern might indeed correspond to splicing speckles, we crossed the GFP‐VIR plants with lines stably expressing splicing protein SR34 fused to RFP (Lorković *et al*., 2008). The observed co‐localization of SR34‐RFP and GFP‐VIR indicates that the GFP‐VIR can localize to splicing speckles (Fig. 3d). Finally, the expression pattern of each protein associating with VIR, MTB (Fig. 3e), FIP37 (Fig. 3f) and HAKAI (Fig. 3g) ‐mCherry overlapped with GFP‐VIR in all tissues examined in *Arabidopsis* root tips (Figs 3e–g, S4).

The merged fluorescent signals in each combination examined reveal that these proteins are found within close proximity in both dividing and differentiated *Arabidopsis* cells. This is consistent with the *in vivo* interaction proteomics data and suggests that they interact functionally and physically.

### Establishing a set of stable transgenic lines that perturb the expression of the proteins associated with m^6^A writing

**Figure 4:**
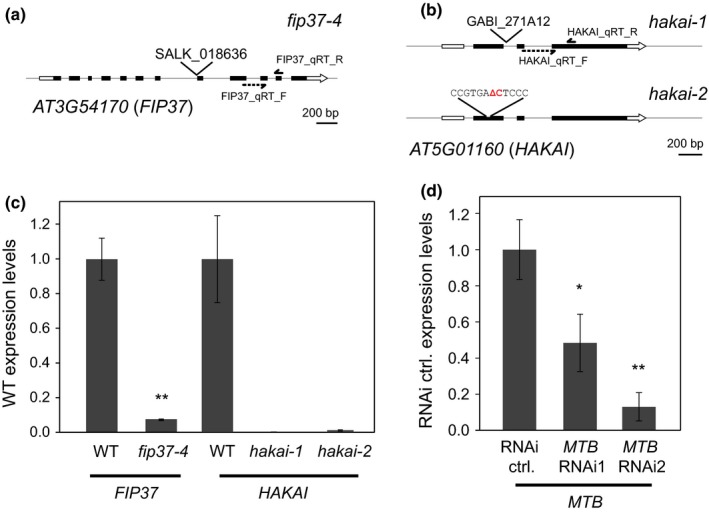
Molecular properties of transgenic lines with depleted expression of proteins associated with *Arabidopsis thaliana* writer proteins. (a) T‐DNA insertion mutant line SALK_018636 is a weak *fip37‐4* allele. (b) *hakai‐1* (GABI_271A12) is an insertional and *hakai‐2* is a CRISPR/Cas9‐derived 1‐bp deletion allele in the *HAKAI* gene. (c) *fip37‐4, hakai‐1* and *hakai‐2* show strongly depleted levels of their respective transcripts as determined by quantitative reverse transcription polymerase chain reaction (qRT‐PCR), in *hakai* alleles, the amounts of *HAKAI* transcript dropped below detection limits of the instrument. (d) Reduced expression of *MTB* was also observed in inducible *UBQ10prom: XVE*>>*MTB* RNAi lines. Data are means ± SE; *, *P *<* *0.05; **, *P *<* *0.01 by ANOVA. The positions of the primer binding sites are depicted on the gene diagrams, where applicable. For the *MTB* RNAi line and the corresponding control, plant material was grown on media supplemented with 5 μM β‐estradiol.

In order to test the requirement for each of the associating proteins in writing m^6^A, we generated a collection of *Arabidopsis* lines defective in the expression of MTB, FIP37, VIR and HAKAI (Figs 1b–d, 4). Null mutations in *MTA* are embryonic lethal (Zhong *et al*., 2008), as are null alleles of *Arabidopsis MTB*,* FIP37* and *VIRILIZER* (Tzafrir *et al*., 2004; Vespa *et al*., 2004). However, we identified a viable hypomorphic allele of *FIP37*, caused by a T‐DNA insertion within its 7^th^ intron (*fip37‐4*, identical to that described in Shen *et al*. (2016); Fig. 4a). We also identified an insertion mutant of *hakai* (*hakai‐1*; Fig. 4b), and generated a deletion *hakai* allele using CRISPR/Cas9 to remove a single nucleotide in the coding sequence of the 1st exon (*hakai‐2*, Fig. 4b). All of these homozygous mutants were viable in our growth conditions. qRT‐PCR confirmed that expression of the respective genes was compromised in each case (Fig. 4c).

We also constructed inducible RNAi lines (Mähönen *et al*., 2014) to knockdown the expression of *MTB* and used RT‐qPCR to confirm that expression was knocked‐down upon addition of the inducer β‐estradiol (Fig. 4d). In summary, we have developed a set of viable genotypes that partially or completely disrupt the expression of genes encoding each of the putative m^6^A writers. These provide a genetic resource to analyse the functional role of MTB, FIP37, VIR and HAKAI in writing m^6^A.

### HAKAI and putative *Arabidopsis* m^6^A writers are required for methylation of mRNA

**Figure 5:**
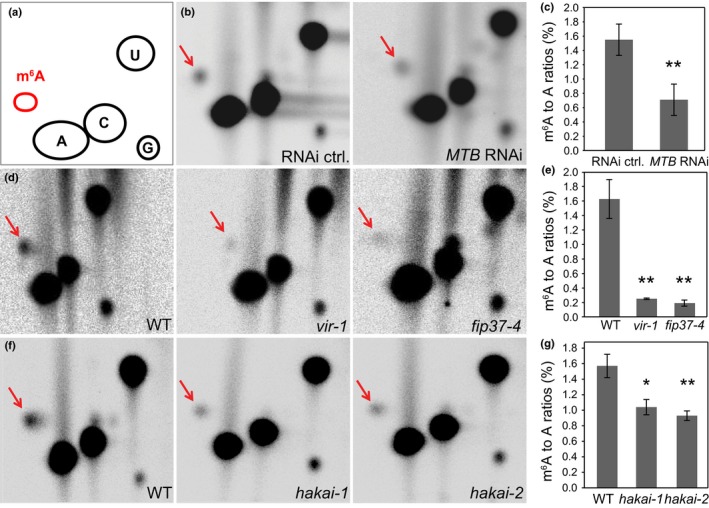
Proteins associating with the *Arabidopsis thaliana* m^6^A writers are required for methylation of mRNA. (a) Schematic positions of the m^6^A and nonmodified nucleotides separated by 2D thin‐layer chromatography. (b, c) m^6^A levels are reduced in poly(A)^+^ RNA of the inducible *UBQ10prom:XVE*>>*MTB* RNAi lines as shown on the 2D thin‐layer chromatogram (b) and quantified from two independent experiments (c). (d, e) Reduced amounts of m^6^A are also seen in poly(A)^+^ RNA of hypomorphic alleles *fip37‐4* and *vir‐1*. (e) The quantification of three independent replicates carried out on *fip37‐4* and *vir‐1*. (f, g) The levels of m^6^A are depleted in poly(A)^+^ RNA isolated from *hakai* mutants, as documented by the chromatograms (e) and assessed by three independent replicates for each mutant (g). Data are means ± SE; *, *P *<* *0.05; **, *P *<* *0.01 by ANOVA. For the *MTB* RNAi line and the corresponding control, plant material was grown on media supplemented with 5 μM β‐estradiol. Location of the m^6^A spot (b, d, f) is indicated by arrows.

We next examined the requirement of each protein factor in writing m^6^A to *Arabidopsis* mRNA. We therefore measured the levels of m^6^A in each of the lines. Following two rounds of poly(A)^+^ RNA purification, mRNA was digested with RNase T1 (which cleaves after every G residue). Fragments were then end‐labelled using [γ‐^32^P]ATP and digested to mononucleotides. These were separated and the m^6^A : A ratios determined as described previously (Fig. 5a; Zhong *et al*., 2008). We found that levels of m^6^A were reduced to 5–15% WT levels in the *fip37‐4*, and *vir‐1* lines and by 50% in the MTB RNAi line after β‐estradiol treatment (Fig. 5b–e). Furthermore, in both *hakai* mutants m^6^A levels were reduced by 35% (Fig. 5f,g). We therefore conclude that each of the newly identified factors, MTB, FIP37, VIR and HAKAI, is required to write WT levels of m^6^A in *Arabidopsis* mRNA.

### Genetic depletion of total m^6^A pools leads to similar phenotypic consequences

**Figure 6:**
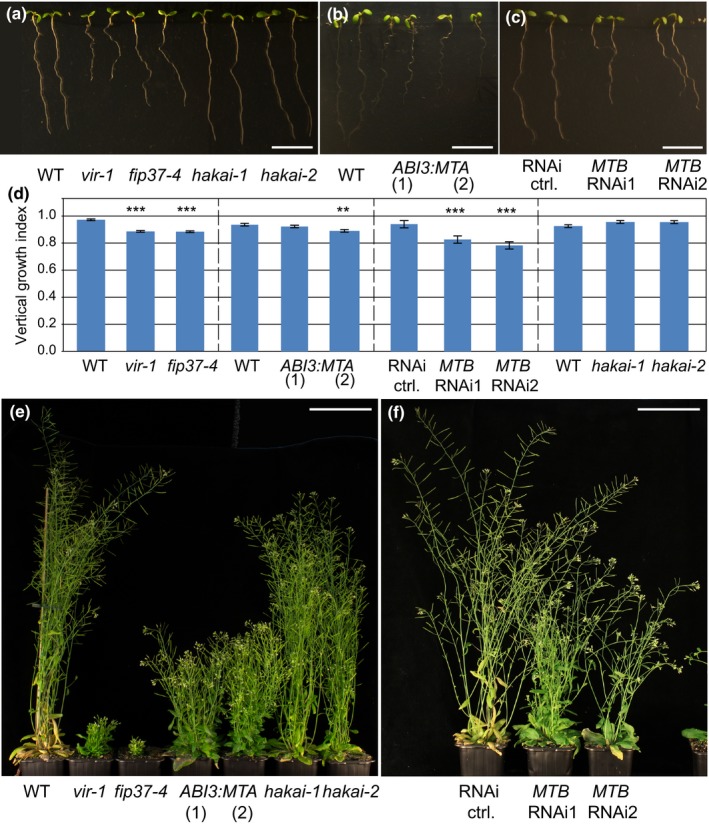
*Arabidopsis thaliana* lines with defective expression of m^6^A writer proteins exhibit similar phenotypes at the macroscopic level. (a–d) The seedlings of hypomorphic lines *fip37‐4*,* vir‐1* (a) and *mta ABI3prom:MTA* (two independent lines, marked as *ABI3:MTA*) (b), as well as two independent inducible *UBQ10prom:XVE*>>*MTB* RNAi lines (c) show similar defects in overall root morphology, whereas both *hakai‐1* knockdown and *hakai‐2* knockout root growth resemble wild‐type (WT) (a); similar trends were seen with in their gravitropic response quantified by vertical growth index (d). (e) Seven‐week‐old plants with strongly depleted levels of m^6^A show altered generative growth, which includes bushy phenotype and reduced apical dominance, whereas *hakai* mutants rather resemble WT controls. (f) Seven‐week‐old *UBQ10prom:XVE*>>*MTB* plants sprayed with 20 μM β‐estradiol also show affected generative growth. Bars: (a–c) 10 mm; (e, f) 10 cm. Data are means ± SE; **, *P *<* *0.01; ***, *P *<* *0.001 by ANOVA. For the *MTB* RNAi seedlings and the corresponding control, plant material was grown on media supplemented with 5 μM β‐estradiol.

**Figure 7:**
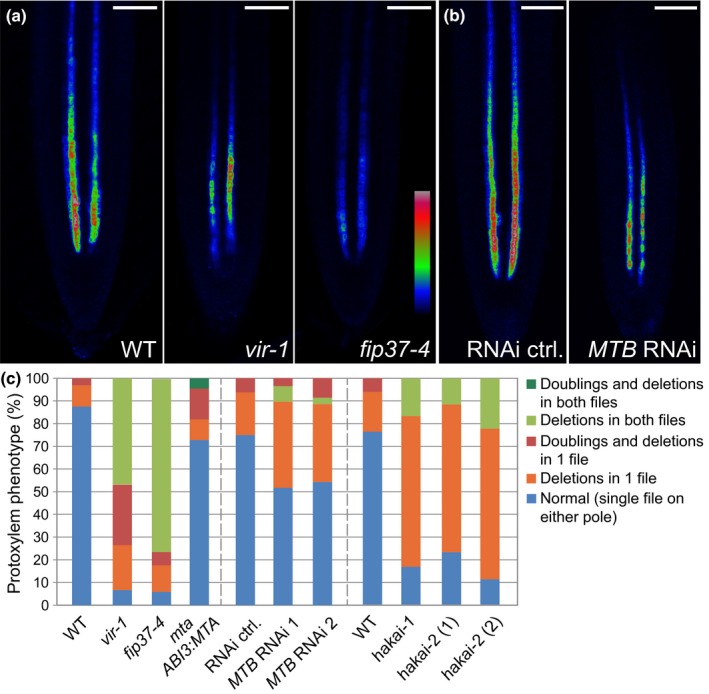
Reduced expression of proteins associating with the *Arabidopsis thaliana* m^6^A writers results in vascular defects. (a, b) Similar to *vir‐1*,*AHP6prom:GFP* fluorescence signal is reduced in the weak *fip37‐4* allele (a) and following induction of inducible *UBQ10prom:XVE*>>*MTB* RNAi (b). (c) Mutants with reduced m^6^A levels show vascular defects, which includes interruptions or duplications of protoxylem files across the primary root. Bars, 50 μm. For the *MTB* RNAi line and the corresponding control, plant material was grown on media supplemented with 5 μM β‐estradiol.

If MTA, MTB, FIP37, HAKAI and VIR function together to write m^6^A, then it would be expected that mutants defective in these components might share similar developmental defects. Indeed, the *mta, mtb, fip37* and *vir* null alleles arrest at the globular stage of embryonic development (seedgenes.org; Tzafrir *et al*., 2004), but both *hakai* alleles are viable (Fig. 6a). Hypomorphic mutants and knockdown lines of these factors or null *mta* mutants rescued by embryo‐specific expression of MTA driven by the *ABI3* promoter (*mta ABI3prom:MTA*) (Bodi *et al*., 2012), show reduced root growth and aberrant gravitropic responses (Fig. 6a–d), whereas both *hakai* alleles rather resemble WT. This distinction may reflect the different impact on global m^6^A levels of these lines, because in each hypomorphic mutant of *MTA* (Bodi *et al*., 2012), *FIP37* and *VIRILIZER*, m^6^A levels were reduced to a greater extent than in both *hakai* alleles (Fig. 5). In addition, the lines with reduced *MTA, MTB, FIP37* and *VIR* expression also show delayed development and reduced apical dominance in the generative phase of development, whereas *hakai* mutants more closely resembled WT with respect to these traits (Fig. 6e,f). Because we isolated the viable *vir‐1* allele in the course of a screen for factors mediating the regulation of *AHP6prom:GFP* in early stages of root development, we also looked at these traits in more detail. Like the *vir‐1* allele which shows a misexpression of *AHP6prom:GFP*, we found that *MTB* knockdown lines and *fip37‐4* mutants also showed reduced expression of the *AHP6prom:GFP* reporter (Fig. 7a,b). Importantly, all mutant and knock‐down lines show defects associated with vascular development. Similar to *vir‐1*, each line exhibits defective protoxylem development, with increased occurrence of interruptions in, and doublings of, protoxylem strands being detected compared to WT (Fig. 7c). In conclusion, all mutants share defects in vascular development and reductions of m^6^A. Embryonic lethality of null alleles and other developmental defects are shared in hypomorphic lines defective in MTA, MTB, FIP37 and VIR function, but these are generally less pronounced in *hakai* mutants.

### *hakai* mutants act synergistically with other m^6^A writer mutants

**Figure 8:**
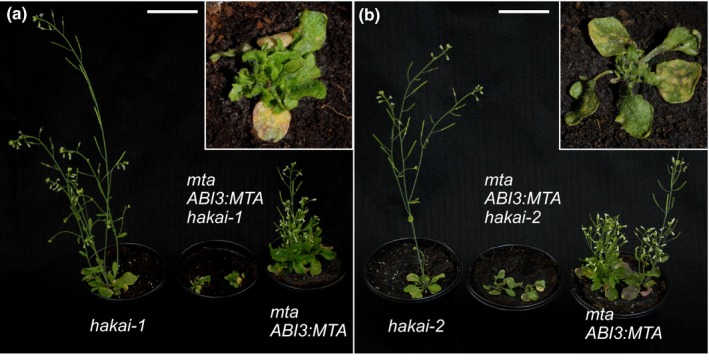
*Arabidopsis thaliana* plants homozygous for either (a) *hakai‐1* or (b) *hakai‐2* crossed with *mta ABI3prom:MTA* show more severe developmental defects than either parent on its own (double mutants magnified in inset). Bars, 5 cm.

Given the relatively weak *hakai* mutant phenotypes compared to the severe developmental defects seen in the hypomorphs of the other m^6^A writer‐associated proteins, we sought to test whether HAKAI also interacted genetically with other writer components. First, we crossed both *hakai‐1* and *hakai‐2* to the *mta ABI3prom:MTA* line (Bodi *et al*., 2012) and selected double homozygous mutant plants from the subsequent filial generations. In both cases, the introduction of the *hakai* mutant into the *mta ABI3prom:MTA* hypomorph background gave rise to plants with a compromised growth phenotype that was far more severe than either parent (Fig. 8). m^6^A levels showed a further slight reduction in m^6^A relative to the *mta ABI3prom:MTA* parent (Fig. S5). Next we crossed the *hakai‐2* mutant to the *fip37‐4* hypomorph and selected plants that were homozygous for *fip37‐4* and heterozygous for *hakai‐2* from the F_2_ generation. These plants were selfed and the F_3_ seed planted on Murashige & Skoog media and the emerging seedlings genotyped with respect to the *hakai‐2* mutation. Of 73 progenies, 23 (31.5%) were found to be WT with respect to *hakai‐2* and 50 (68.5%) were heterozygous. No homozygous *hakai‐2* seedlings were found. This suggests that the combination of the *fip37‐4* and *hakai‐2* mutations is lethal; indeed the WT:heterozygotes observed ratio is close to the 1:2 that would be predicted if this were the case. We did not observe an increase in the number of nongerminating seeds, suggesting that the double mutants aborted very early in their development.

## Discussion

### Role of m^6^A in plant development

A regulatory role of *N*6‐adenosine methylation (m^6^A) in development and in determining cell fate has been demonstrated in major model systems such as mouse, *Drosophila melanogaster* and *Saccharomyces cerevisiae* (Clancy *et al*., 2002; Hongay & Orr‐Weaver, 2011; Geula *et al*., 2015; Haussmann *et al*., 2016). m^6^A has recently been shown to have a crucial role during murine stem cell development, where it prompts stem cell differentiation from their naïve stage (Geula *et al*., 2015). *Arabidopsis* full knockouts of MTA, MTB, FIP37 and VIR also do not progress past the embryonic globular stage (Tzafrir *et al*., 2003; Vespa *et al*., 2004; Zhong *et al*., 2008). We have circumvented this embryo lethality by establishing a collection of hypomorphic, mutants or RNAi lines for each of the proteins associated with the m^6^A writer complex in order to study the effects of reduced m^6^A in postembryonic development. In a recently published study, (Shen *et al*., 2016) reported that reduced FIP37 expression resulted in increased proliferation of the shoot apical meristem. Our findings independently expand this, further revealing that impaired expression of any of the five m^6^A writer components we identify results in a range of developmental defects, including vascular formation, implying a role for m^6^A in establishing and maintaining these processes.

### Conserved Eukaryotic writer proteins and role of VIRILIZER

Based on our genetic and biochemical data, we propose that the core constituents of the plant writer complex are nuclear localized proteins MTA, MTB, FIP37, VIR and HAKAI. Orthologues of MTA and MTB interact with each other and with orthologues of FIP37 in *S. cerevisiae* (Agarwala *et al*., 2012) and human cells (Liu *et al*., 2014; Ping *et al*., 2014; Schwartz *et al*., 2014; Wang *et al*., 2014b). In *S. cerevisiae*, writing of m^6^A requires orthologues of MTA (IME4), FIP37 (MUM2) and the yeast‐specific SLZ1 (Agarwala *et al*., 2012). In mammals, in addition to the orthologues of MTA (METTL3) and FIP37 (WTAP), MTB (METTL14) and the splicing/methylation factor VIR (KIAA1429) are also present in the complex and required for m^6^A writing. *Arabidopsis* MTA and MTB are nonredundant and, similar to METTL3 and METTL14 (Liu *et al*., 2014; Ping *et al*., 2014; Wang *et al*., 2014b), they also interact directly in Y2H.

In contrast to *S. cerevisiae*, where the m^6^A writers reside in nucleoli (Agarwala), their plant orthologues occupy the nucleoplasm, similar to animal systems (Bokar *et al*., 1997; Niessen *et al*., 2001; Zhong *et al*., 2008; Liu *et al*., 2014; Ping *et al*., 2014). Subnuclear domains, so called nuclear speckles, correspond to sites with active transcription in the interchromatin regions. They are connected with the presence of splicing factors, but less with the accumulation of other factors participating on RNA processing in microscope localization studies (Spector & Lamond, 2011; Reddy *et al*., 2012). Analogously to their animal orthologues (Liu *et al*., 2014; Ping *et al*., 2014), plant writers also show a similar punctate pattern (Zhong *et al*., 2008) and co‐localize with the splicing factor SR34‐RFP. This splicing factor, as well as the co‐localizing writers, shows a more diffuse pattern in the meristematic cells, whereas in the cells above the elongation zone the speckle pattern is more prevalent. This likely coincides with differential transcriptional activities in rapidly dividing and differentiated cells, similar to observations from animal systems (Tillemans *et al*., 2006; Spector & Lamond, 2011; Reddy *et al*., 2012). Because m^6^A affects splicing in mammals and *D. melanogaster* (Liu *et al*., 2014; Ping *et al*., 2014; Alarcón *et al*., 2015a; Haussmann *et al*., 2016; Lence *et al*., 2016) possibly by recruiting splicing factors (Xiao *et al*., 2016), it is interesting to speculate whether m^6^A could also regulate splicing in plants. The rather normal splicing patterns observed in *vir‐1* root tip suggests that m^6^A is not involved in large‐scale regulation of splicing in plants or that splicing regulation only occurs at the transcript or tissue‐specific level, which is below the detection limit of the root‐tip RNA‐Seq.

In yeast, mRNA methylation only occurs naturally under very specific conditions where cells must be diploid and starved for both a nitrogen and fermentable carbon source (Clancy *et al*., 2002). In plants and mammals, mRNA methylation amounts vary between different developmental stages or organ types (Zhong *et al*., 2008; Meyer *et al*., 2012), indicating that regulation in these multicellular organisms is likely to be more subtle than a simple on or off state. It seems that variations of the writer complex evolved in other eukaryotes. According to our *in silico* analysis, VIR and HAKAI are missing in the genomes of *Fungi*, including *S. cerevisiae*. VIR has been implicated in the regulation of sex‐specific alternative splicing in *D. melanogaster* (Hilfiker *et al*., 1995; Niessen *et al*., 2001), but more recently it was also found to be required for m^6^A formation in human cells (Schwartz *et al*., 2014). In *D. melanogaster*, the FIP37 homologue Fl(2)D closely associates with VIRILIZER (Ortega *et al*., 2003) and interacts genetically with methylation pathways to regulate sex‐specific splicing events (Haussmann *et al*., 2016; Lence *et al*., 2016). In human cells their orthologues WTAP and KIAA1429 also co‐purify (Horiuchi *et al*., 2013; Schwartz *et al*., 2014). Our data thus indicate that writer proteins in *Arabidopsis* more closely resemble those in mammals than yeast. Our results also highlight the conserved role of VIRILIZER, although whether this large protein is performing a scaffolding role or is catalytically active (and/or carries out a regulatory function) remains to be tested.

### HAKAI is a new element required for the function of the *Arabidopsis* m^6^A writer complex and directly interacts with its core components

Mammalian HAKAI, also known as Casitas B‐lineage lymphoma‐transforming sequence‐like protein 1 (CBLL1), was initially identified as an E3 ubiquitin ligase that facilitates endocytosis of E‐cadherin at cell–cell contacts, thus regulating epithelial integrity (Fujita *et al*., 2002). More recently, it has also been implicated with influencing RNA–protein interactions in animal systems (Fujita *et al*., 2002; Figueroa *et al*., 2009) and has been reported in association with m^6^A writers (Horiuchi *et al*., 2013), but a role in m^6^A writing has not been proposed until now. Although believed to act on E‐cadherin complex at the plasma membrane, human and *D. melanogaster* HAKAIs are localized predominantly in the nucleus and in lower amounts at the plasma membrane and in the cytoplasm (Figueroa *et al*., 2009; Kaido *et al*., 2009). *Arabidopsis* HAKAI‐mCherry resides mainly in the nucleus (Fig. 3g). Plants lack cadherins and the mechanisms determining cell polarity are generally different in metazoans and plants (Kania *et al*., 2014). Thus, the RNA‐associated m^6^A‐forming function of HAKAI that we identify here is likely to prevail in *Arabidopsis*. As phenotypes of *hakai* knockouts are rather subtle compared to the embryo lethality of other complex member knockouts, it is possible that HAKAI may have a more complex role. However, the extreme severity of the *hakai mta ABI3prom:MTA* and the lethality of the *hakai fip37‐4* double mutants supports the conclusion that HAKAI is a *bona fide* functional member of the methylation complex. It also suggests that as methylation amounts drop below ~ 10% of wild‐type (WT), the severity of growth phenotypes increases dramatically. The *mta ABI3prom:MTA* line was generated by complementing an *MTA* insertion knockout with an *MTA* coding sequence under control of the *ABI3* promoter, which drives high expression, and full m^6^A methylation, in seeds, but has very low activity post‐germination (Bodi *et al*., 2012). Thus, the combination of this genotype with *hakai* knockout would only be expected to show a double mutant phenotype post‐germination. By contrast, the *fip37‐4* hypomorph shows low levels of *FIP37* expression and has poor seed set even before combining with other mutant genotypes. Thus, selfing a plant that is homozygous for *fip37‐4* and heterozygous for *hakai‐2* would be expected to give a further, possibly developmentally lethal, reduction in m^6^A levels in the early stages of embryonic development.

HAKAI has been extensively studied in mammals due to its connection with oncogenesis (Aparicio *et al*., 2012), primarily attributed to its controlling of cell–cell adhesion (Fujita *et al*., 2002). However, it has been reported that this protein, through an uncharacterized mechanism, interacts with **p**olypyrimidine tract binding protein associated **s**plicing **f**actor (PSF) and promotes its ability to bind specific (particularly tumourigenesis related) transcripts (Figueroa *et al*., 2009). Mammalian HAKAI specifically ubiquitinates phosphotyrosine modified E‐cadherin (Fujita *et al*., 2002) and it may be that a similar mechanism is required for its activity within the m^6^A writer complex. Given the association of this protein with the writer complex in both plants and animals, and considering that in plants it is required for normal m^6^A methylation amounts, we propose that HAKAI is likely to also play a similar role in mRNA methylation in mammals. In this context, our findings could improve understanding of the mechanistic role of HAKAI in promoting tumorigenesis and regulating the binding of PSF with cancer‐associated mRNA transcripts.

## Author contributions

K.R., M.Z., A.C., Z.B., M.K., M.S., H.L. and S.Z. conducted experiments; A.C. performed genetic screen; D.E. and G.D.J. made the TAP experiment; S.E‐S. and N.P.M. carried out bioinformatics analyses; K.R., J.H., R.G.F. and Y.H. conceived research and designed experiments; and K.R. and R.G.F wrote the manuscript. All authors read and commented on the final version of the manuscript.

## Supporting information

Please note: Wiley Blackwell are not responsible for the content or functionality of any Supporting Information supplied by the authors. Any queries (other than missing material) should be directed to the *New Phytologist* Central Office.

**Fig. S1** Sequence conservation of VIR homologues.
**Fig. S2 **Additional molecular and phenotypic characterization of *vir‐1*.
**Fig. S3** Sequence conservation of HAKAI homologues.
**Fig. S4 **Expression of VIR and other proteins associated with m^6^A writing in the root tip.
**Fig. S5 **m^6^A levels are reduced in adult *hakai‐2*,* mta ABI3prom:MTA* and their double mutant combination.
**Table S1 **Summary of oligonucleotides used in this study
**Table S6 **List of genes involved in vascular formation, which show altered expression in the *vir‐1* root tips
**Table S7 **TAP‐VIR proteomics data

**Table S2 **Summary of splicing events altered in the *vir‐1* background

**Table S3 **List of genes with significantly changed expression in the *vir‐1* root tips as determined by the DESeq software package

**Table S4 **Summary of gene ontology (GO) analysis of genes misexpressed in the *vir‐1* mutants.

**Table S5 **Establishing list of genes required for vascular formation
