# Supplementary material for: Identification of factors required for m6A mRNA methylation in Arabidopsis reveals a role for the conserved E3 ubiquitin ligase HAKAI
Source: New Phytol. 2017 May 15;215(1):157–72. doi: 10.1111/nph.14586 (PMC5488176; doi:10.1111/nph.14586)
Supplement: Supplementary file 1 — Fig. S1 Sequence conservation of VIR homologues. Fig. S2 Additional molecular and phenotypic characterization of vir‐1. Fig. S3 Sequence conservation of HAKAI homologues. Fig. S4 Expression of VIR and other proteins associated with m6A writing in the root tip. Fig. S5 m6A levels are reduced in adult hakai‐2, mta ABI3prom:MTA and their double mutant combination. Table S1 Summary of oligonucleotides used in this study Table S6 List of genes involved in vascular formation, which show altered expression in the vir‐1 root tips Table S7 TAP‐VIR proteomics data [file NPH-215-157-s001.pdf]

New Phytologist

New Phytologist Supporting Information

Article title: Identification of Factors Required for m^{6}A mRNA Methylation in *Arabidopsis* Reveals a Role for the Conserved E3 Ubiquitin Ligase HAKAI

Authors: Kamil Růžička, Mi Zhang, Ana Campilho, Zsuzsanna Bodi, Muhammad Kashif, Mária Saleh, Dominique Eeckhout, Sedeer El-Showk, Hongying Li, Silin Zhong, Geert De Jaeger, Nigel P. Mongan, Jan Hejátko, Ykä Helariutta, Rupert G. Fray

Article acceptance date: 19 March 2017

The following Supporting Information is available for this article:

Fig. S1 Sequence conservation of VIR homologues.

Fig. S2 Additional molecular and phenotypic characterisation of *vir-1*.

Fig. S3 Sequence conservation of HAKAI homologues.

Fig. S4 Expression of VIR and other proteins associated with m^{6}A writing in the root tip.

Fig. S5 m^{6}A levels are reduced in adult *hakai-2*, *mta ABI3prom:MTA* and their double mutant combination.

Table S1 Summary of oligonucleotides used in this study.

Table S2 Summary of splicing events altered in the *vir-1* background.

Table S3 List of genes with significantly changed expression in the *vir-1* root tips, as determined by the DESeq software package.

Table S4 Summary of Gene Ontology (GO) analysis of genes misexpressed in the *vir-1* mutants.

Table S5 Establishing list of genes required for vascular formation.

Table S6 List of genes involved in vascular formation, which show altered expression in the *vir-1* root tips.

Table S7 TAP-VIR Proteomics Data.

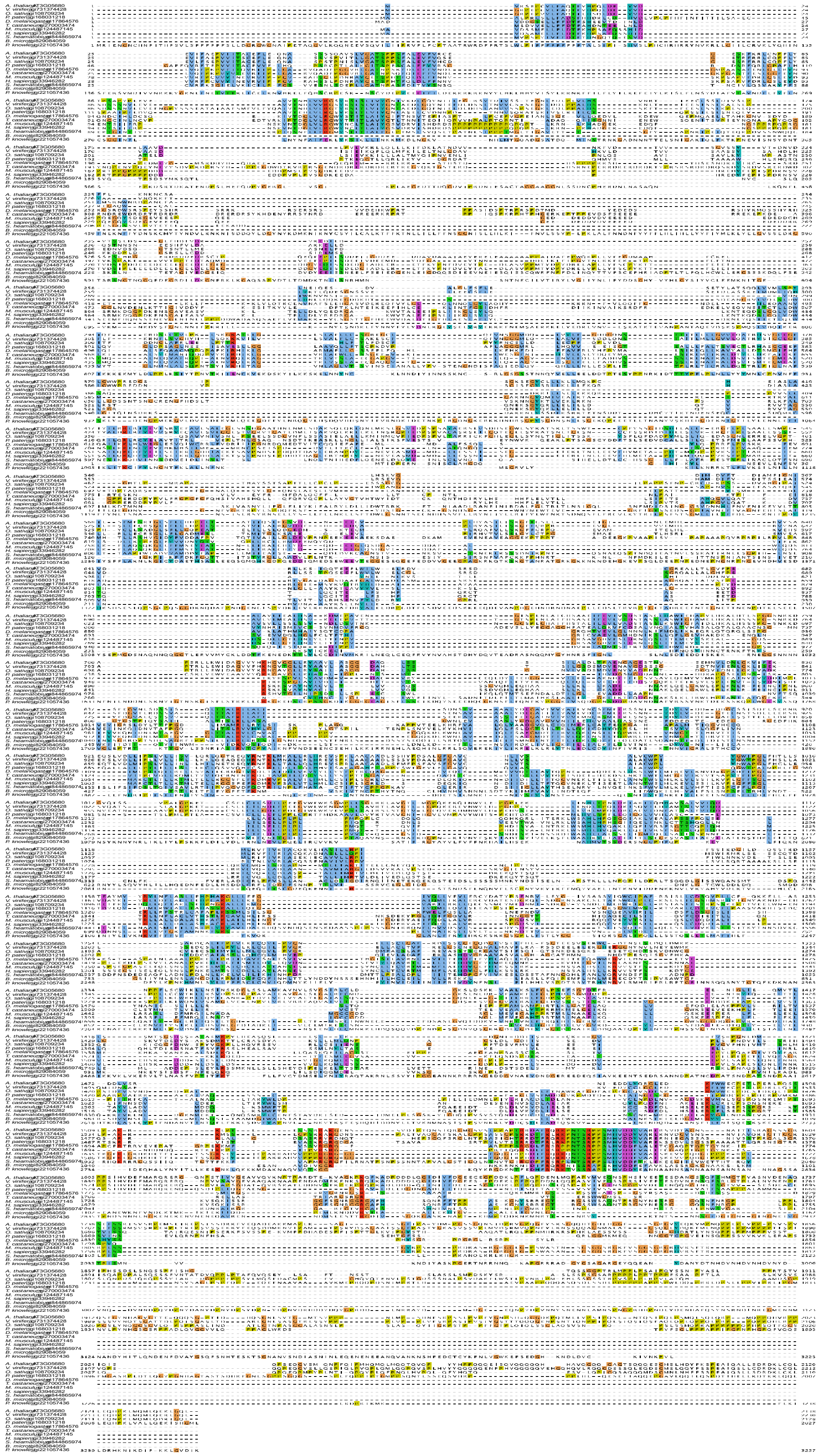

Fig. S1 Sequence conservation of VIR homologues. Multiple sequence alignment of sequences related to *Arabidopsis* VIR, including *D. melanogaster* Virilizer and human KIAA1429.

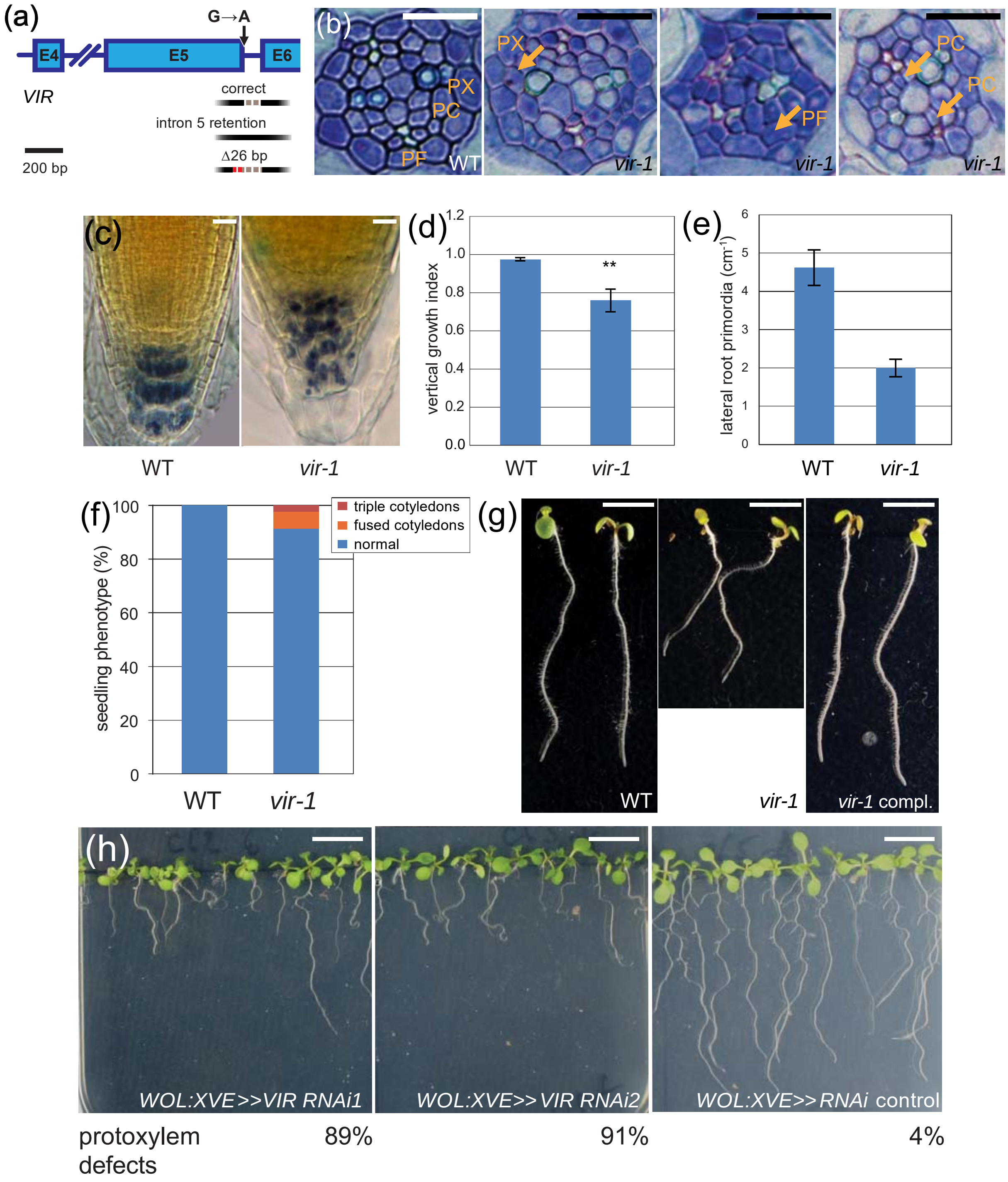

Fig. S2 Additional molecular and phenotypic characterisation of *vir-1*. (a) Sequencing of major transcripts caused by *vir-1* mutation indicates that, whilst defectively processed *VIR* mRNAs with a predicted frame shift and/or premature stop codon predominate, a small proportion of *VIR*

transcripts remain correctly processed. (b) Cross sections of *vir-1* primary root; compared to wild type (WT), *vir-1* shows multiple vascular defects, in protoxylem (PX), protophloem (PP) and also (pro)cambium (PC), as shown on each representative image. (c) *vir-1* shows defects in root cap formation; columella starch granules were stained by Lugol solution. (d-g) *vir-1* exhibits defective gravity response (d), lateral root formation (e) and also overall seedling phenotype (f); the *vir-1* seedling phenotype can be rescued by *VIRprom:GFP-VIR* transgene (g). (h) The phenotype of *vir-1* seedlings can be phenocopied by inducible *VIR* RNAi lines. Data are means ± SE. Bars: 20 µm in (b) and (c), 5 mm in (g), and 10 mm in (h). For the *VIR* RNAi line and the corresponding control, plant material was grown on media supplemented with 5 µM β-estradiol.

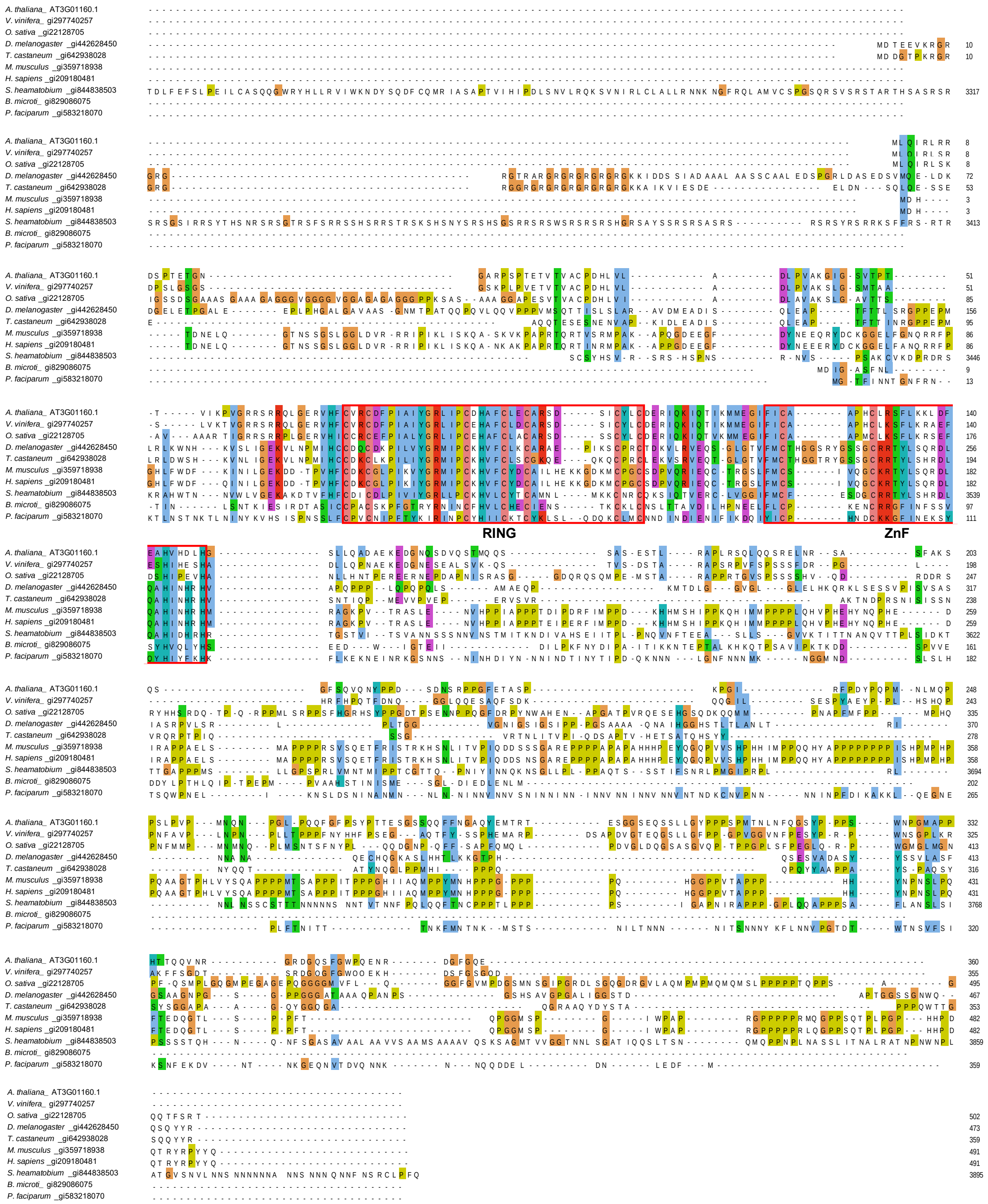

Fig. S3 Sequence conservation of HAKAI homologues. Multiple sequence alignment of sequences related to *Arabidopsis* HAKAI, including *D. melanogaster* and human HAKAI. Conserved RING and zinc finger (ZnF) domains are boxed in red.

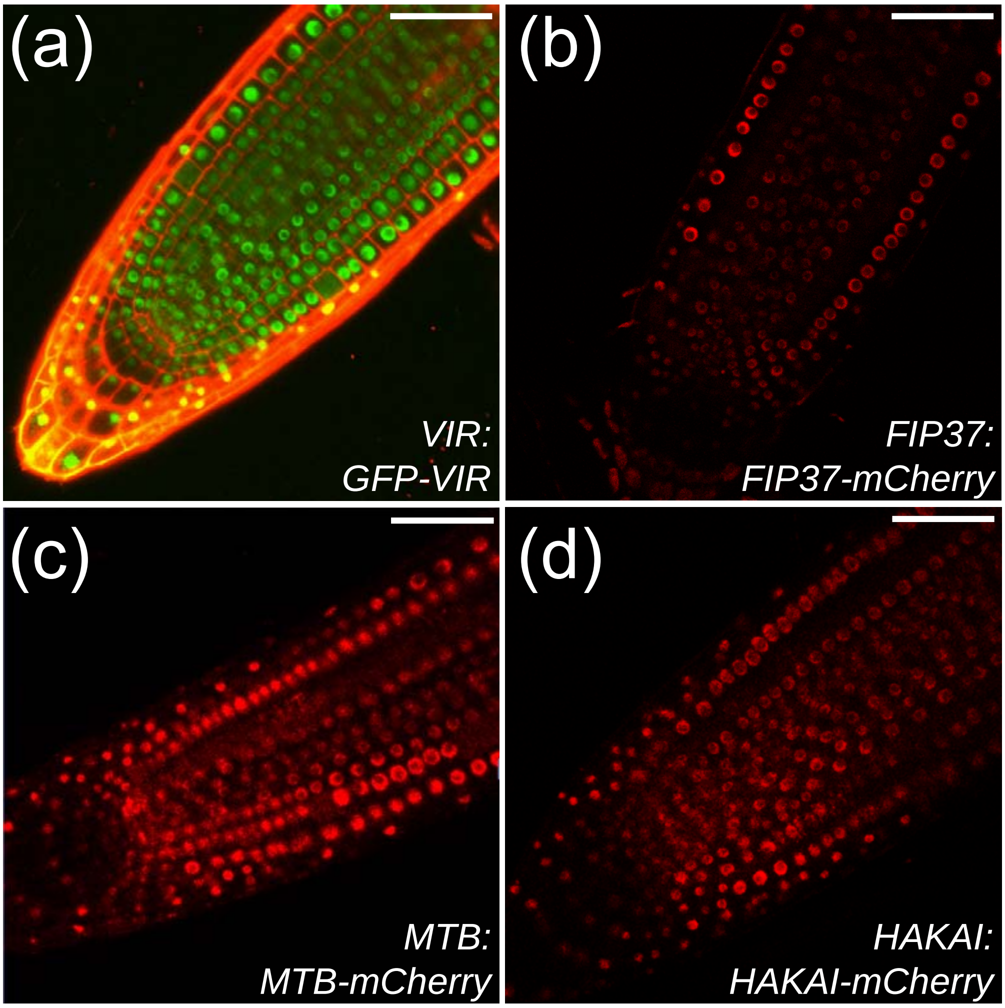

Fig. S4 Expression of VIR and other proteins associated with m^{6}A writing in the root tip. (a-d) *VIRprom:GFP-VIR* translational fusion (a) shows a similar subcellular localisation and expression pattern as FIP37- (b), MTB- (c) and HAKAI-mCherry (d) proteins expressed under their native promoters. Bars: 50 µm.

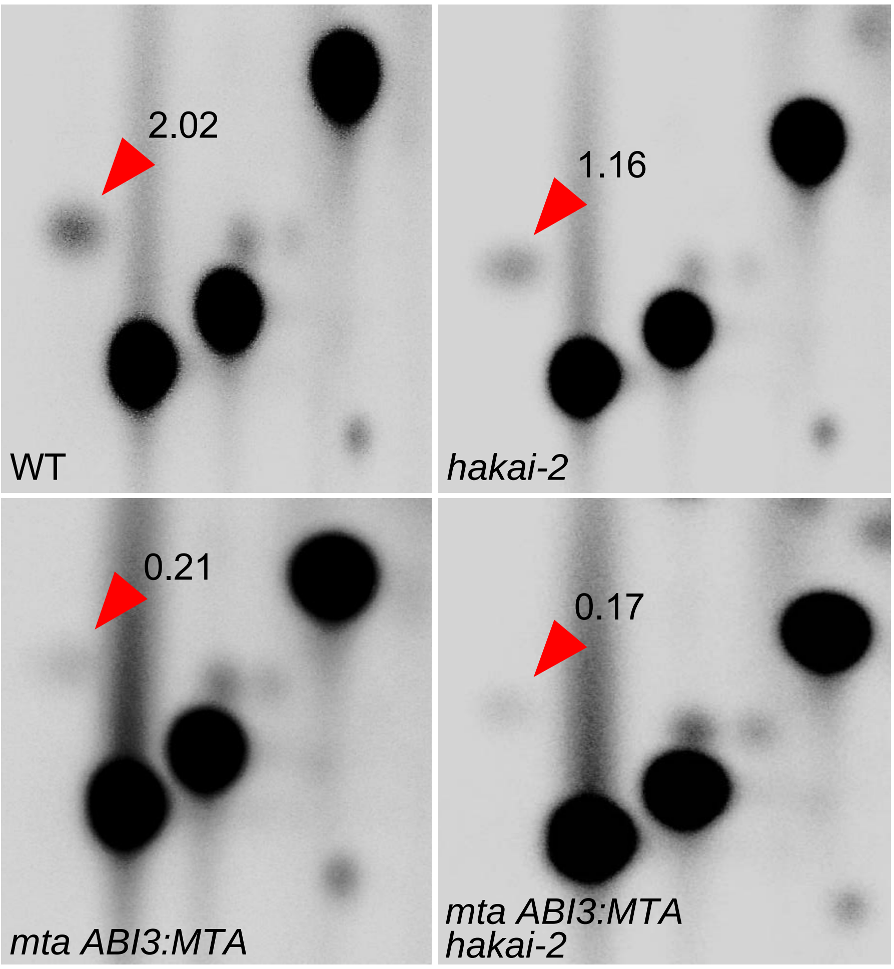

Fig. S5 m^{6}A levels are reduced in adult *hakai-2*, *mta ABI3prom:MTA* and their double mutant combination. Percentages of m^{6}A relative to the adenines are indicated.

New Phytologist

Table S1 Summary of oligonucleotides used in this study.

Genotyping	
line	*fip37-4*
FIP37-4_F	ATCGCAAAGAGAAAAGAAGCG
FIP37-4_R	GTTCTGCACTTTGCCATAAGC
SALK_LBb1 (+FIP37-4_R)	gcgtggaccgcttgctgcaact
line	*hakai-1*
HAKAI-1_F	ATGCTTCAAATTCGTCTTCGG
HAKAI-1_R	CTCCTGCCCAAAGCCATCACG
Gabi_o3269 (+HAKAI-1_R)	GAAGGCGGGAAACGACAATCTG
(q)RT-PCR	
VIR_E4_F	TTCTTGAATCAGAGACTTATTTGGC
VIR_E6_R	TTAGAGAGACCTTCCAGTGC
MTB_qPCR_F	GTACTGTGTTTCAGCGTTCC
MTB_qPCR_R	TTCTGAGTCGAACCATAAGGAG
FIP_qPCR_R	GTCAATGCAGGCGAGGAGGT
FIP_qPCR_F	ACTGCTGCAATGTTGTCCTGGAG
VIR_qPCR_F	ACGCAAGTCCAGCCTTACTATCAC
VIR_qPCR_R	CGGTCACTTAATAGAGCCTGAATGG
HAKAI_qPCR_F	CCACCGTAATCAAGCCCGTC
HAKAI_qPCR_R	ATCCGCTCATCACATAGATAGCAG
UBQ10_qPCR_F	CACACTCCACTTGGTCTTGCGT
UBQ10_qPCR_R	TGGTCTTTCCGGTGAGAGTCTTCA
Cloning and Y2H	
VIRprom_ApaIF	ATAGGGCCCGCCTGCCAGTTTCGGGCAAACC
VIRprom_XhoIR	ATACTCGAGCATGCCTCCAGGATTGATTGGTG
MTB_prom_F	AAGAGCTCCTATTTTTAACCAATTTGTGGTG
MTB_prom_R	AAGGATTCTTTTTTGCGAAAACTGTGCTACA
FIP37_prom_F	AAAAAGCAGGCTCTATGAAGAAGAAACAAGAAGAGAG
FIP37_prom_R	AGAAAGCTGGGTTATTAAAGCCGTACATGTCAAAAC
HAKAI_prom_F	AAGAGCTCCTTCTTGATATTGGTGTGAAAGAG
HAKAI_prom_R	AAGGATTCTGTCGCTGTAAAAGTTACATG
VIRcds_XhoIF	ATACTCGAGATGGTACGATCTGAGCCTTGTG
VIRcds_AvrIIR	ATACCTAGGTAGCTGCCCAAGTTTTTCCTGTC
MTB_cds_F	AAAAAGCAGGCTCTATGAAGAAGAAACAAGAAGAGAG
MTB_cds_R	AGAAAGCTGGGTTATTAAAGCCGTACATGTCAAAAC
FIP37_cds_F	AAAAAGCAGGCTCTATGGAGTTTTCATCACAAGACG
FIP37_cds_R	AGAAAGCTGGGTTTTCTCCACCAGCAATTTCTTC

HAKAI_cds_F	AAAAAGCAGGCTAAATGCTTCAAATTCGTCTTCGG
HAKAI_cds_R	AGAAAGCTGGGTTCTCCTGCCCAAAGCCATCACG
VIR_cds1/2_F	AAAAAGCAGGCTTAATGGTACGATCTGAGCCTTGTG
VIR_cds1/2_R	AGAAAGCTGGGTTTCAATCAAGATTCCCAGGAGAAACAA
VIR_cds2/2_F	AAAAAGCAGGCTCTATTATCTCCTGCAAAGGTGTT
VIR_cds2/2_R	AGAAAGCTGGGTTTCATAGCTGCCCAAGTTTTTCC
RNAi constructs	
EMB2016_RNAi1_F	TCGAGAAGGCTGCCTCCAATTTGTCAG
EMB2016_RNAi1_R	GATCCTGACAAATTGGAGGCAGCCTTC
EMB2016_RNAi2_F	AACTCGAGAAGTCCAGCCTTACTATCACC
EMB2016_RNAi2_R	GATCCTGACAAATTGGAGGCAGCCTTC
MTA-B_RNAi1_F	AAAGTCGACTTCCCCCAAATCTAAAACCC
MTA-B_RNAi1_R	AAAAGATCTACGCTTCTTTTCTCTTTGCG
MTA-B_RNAi2_F	AAAGTCGACGCCTGGTGTTAGAACTGGGA
MTA-B_RNAi2_R	AAAAGATCTACTGCTGCAATGTTGTCCTG
FIP37_RNAi1_F	AACTCGAGATGAAGCTGGTGAAGCCAGT
FIP37_RNAi1_R	AAGGATCCGGCCCTCCTCTATCAGAACC
FIP37_RNAi2_F	AACTCGAGTGGTGGACCTCCTGGTAAAG
FIP37_RNAi2_R	AAGGATCCTTCACACCTTCTGAAACCCC
HAKAI-like_RNAi1_F	AACTCGAGTGATCATGCCTTTTGCCTT
HAKAI-like_RNAi1_R	AAGGATCCGGCGGTTGCATAAGATTCAT
HAKAI-like_RNAi2_F	AACTCGAGGAAACCCTCTTTCTCAGCC
HAKAI-like_RNAi2_R	AAGGATCCTAGCAGATGGAATCACTGCG

New Phytologist

Table S2 Summary of splicing events altered in the *vir-1* background (see separate Excel file). There were no substantial changes in annotated splicing events in the *vir-1* transcriptome as assessed by the rMATS analysis. The false discovery rate was set as *-c* = 0.0001 for 0.01 % difference. The outputs were filtered by $Q < 0.001$. The types of splicing events are as follows: SE, skipped exon; RI, retained intron; A5S, alternative 5'; A3S alternative 3'; MXE, mutually exclusive exon. Further details to the table are available at http://rnaseq-mats.sourceforge.net/user_guide.htm.

Table S3 List of genes with significantly changed expression in the *vir-1* root tips, as determined by the DESeq software package (see separate Excel file). BaseMean, mean normalised read counts from Control and *vir-1* expression profiles; log2FoldChange, the logarithm (to basis 2) of the fold change; p_val, *P* value for the statistical significance of this change; q_val - *P* value adjusted for multiple testing with the Benjamini-Hochberg procedure (FDR).

Table S4 Summary of Gene Ontology (GO) analysis of genes misexpressed in the *vir-1* mutants. GO terms of biological processes (P), molecular function (F), and cellular component (C) associated with *vir-1* root tip transcriptome as determined by the AgriGO analysis output (see separate Excel file).

Table S5 Establishing list of genes required for vascular formation. Based on the GO terms listed (GO ID, GO term), an arbitrary list of genes essential for vascular development has been generated (see separate Excel file).

Table S6 List of genes involved in vascular formation of *Arabidopsis thaliana*, which show altered expression in the *vir-1* root tips. BaseMean, mean normalised read counts from Control and *vir-1* expression profiles; log2FoldChange, the logarithm (to basis 2) of the fold change; p_val, *P* value for the statistical significance of this change; q_val, *P* value adjusted for multiple testing with the Benjamini-Hochberg procedure (FDR).

**Gene ID**	**Description**	**BaseMean Control**	**BaseMean *vir-1***	**log2FoldChange**	**p_val**	**q_val**
AT1G17840	ABCG11 ATP-binding cassette G11	16.380	300.899	4.199	8.67E-60	6.24E-57
AT3G05680	VIR	9201.683	2746.734	-1.744	2.79E-16	2.17E-14
AT1G14000	VIK VH1-interacting kinase	2240.318	1385.943	-0.693	1.12E-11	4.59E-10
AT4G29810	ATMKK2 MAP kinase kinase 2	969.861	570.845	-0.765	1.69E-09	4.49E-08
AT3G24770	CLE41 CLAVATA3/ESR-RELATED 41	63.045	171.555	1.444	8.00E-09	1.87E-07
AT1G19850	ARF5 AUXIN RESPONSE FACTOR 5	1151.480	1712.581	0.573	1.44E-08	3.22E-07
AT2G20130	LCV1 like COV 1	803.605	496.345	-0.695	7.57E-08	1.44E-06
AT1G78770	APC6 anaphase promoting complex 6	857.480	533.685	-0.684	8.56E-07	1.28E-05
AT5G16560	KAN1 KANADI1	849.072	1230.170	0.535	1.43E-06	2.04E-05
AT5G05340	PRX52 peroxidase 52	2.187	33.875	3.953	2.48E-06	3.32E-05
AT2G20120	COV1 CONTINUOUS VASCULAR RING	961.477	633.039	-0.603	4.39E-06	5.52E-05
AT2G42580	VIT VHI-INTERACTING TPR CONTAINING PROTEIN	2242.737	1623.271	-0.466	9.25E-06	0.000107
AT4G14940	AO1 amine oxidase 1	585.205	370.657	-0.659	1.14E-05	0.000129
AT5G19530	ACL5 ACAULIS 5	517.271	330.206	-0.648	4.65E-05	0.000444
AT4G36890	IRX14 irregular xylem 14	1832.022	1368.449	-0.421	4.94E-05	0.000469
AT1G62360	STM SHOOT MERISTEMLESS	1.112	34.832	4.969	0.000113	0.000974
AT5G66300	VND3 VASCULAR-RELATED NAC-DOMAIN 3	135.257	73.248	-0.885	0.000461	0.003238
AT2G18290	APC10 anaphase promoting complex 10	175.469	105.166	-0.739	0.000668	0.00447
AT1G05630	5PTASE13 inositol-polyphosphate 5-phosphatase 13	813.120	606.877	-0.422	0.000896	0.005767
AT1G70560	TAA1 tryptophan aminotransferase of Arabidopsis 1	1599.973	1188.834	-0.428	0.000932	0.005957
AT5G42080	ADL1 dynamin-like protein	11460.052	9125.038	-0.329	0.001124	0.006971
AT3G55830	EPC1 ECTOPICALLY PARTING CELLS	970.606	733.196	-0.405	0.001206	0.007377
AT5G02030	BLH9 BEL1-LIKE HOMEODOMAIN 9	21.066	53.466	1.344	0.001657	0.009669
AT1G14320	SAC52 SUPPRESSOR OF ACAULIS 52	45414.187	36981.452	-0.296	0.001967	0.011145

AT1G62700	VND5 VASCULAR RELATED NAC-DOMAIN PROTEIN 5	46.811	20.481	-1.193	0.002773	0.014807
AT1G09540	MYB61 MYB DOMAIN PROTEIN 61	478.090	370.934	-0.366	0.003931	0.019749
AT2G01850	EXGT-A3 endoxyloglucan transferase A3	1291.192	1905.240	0.561	0.005267	0.025138
AT2G36570	PXC1 PXY/TDR-correlated 1	194.512	128.362	-0.600	0.006895	0.031376
AT4G07410	PCN POPCORN	4806.347	5588.636	0.218	0.006905	0.031399
AT1G20330	CVP1 COTYLEDON VASCULAR PATTERN 1	9367.933	10844.088	0.211	0.007769	0.034645
AT5G23080	TGH TOUGH	1280.979	1536.257	0.262	0.008531	0.03735
AT1G27320	AHK3 histidine kinase 3	853.294	1165.047	0.449	0.008549	0.037414
AT2G39830	DAR2 DA1-related protein 2	388.860	495.316	0.349	0.00923	0.039781
AT1G22740	ATRABG3B GTPase	376.429	287.132	-0.391	0.009442	0.040533
AT1G80100	AHP6 ARABIDOPSIS HISTIDINE KINASE 6	57.886	48.242	-0.263	0.56665	0.819102

Table S7 TAP-VIR Proteomics Data. Protein Identification details obtained with the 4800 MALDI TOF/TOF[TM] Proteomics analyzer (AB SCIEX) and the GPS explorer v3.6 (AB SCIEX) software package combined with search engine Mascot version 2.2 (Matrix Science) and database TAIR10. **Protein score:** The score calculated by the Mascot search engine for each protein. This score is based on the probability that peptide mass matches are non-random events. If the Protein Score is equal to or greater than the Mascot® Significance Level calculated for the database search, the protein match is considered to be statistically non-random at the 95% confidence interval. Protein score = -10*Log(*P*), where *P* is the probability that the observed match is a random event. **Expect**: Protein score expectation value. **RMS error (ppm):** RMS error of the set of matched mass values, in ppm. **Sequence coverage %:** Percentage of protein sequence covered by assigned peptide matches. **Unique peptides**: The number of peptides with unique sequences matching the selected protein. **Total Ion Score**: A score calculated by weighting Ion Scores for all individual peptides matched to a given protein. **Peptide Number:** Peptide index number within the list of peptides associated with a given protein. **Start**: The starting position of the peptide in the protein. **End:** The ending position of the peptide in the protein. **Observed**: The observed monoisotopic mass of the peptide in the spectrum (m/z). **Mr (Exp)**: The experimental mass of the peptide calculated from the observed m/z value. **Mr (Calc)**: The theoretical mass of the peptide based on its sequence. **Delta (Da)**: The difference between the theoretical (Mr (Calc)) and experimental (Mr (Exp)) masses, in daltons. **Miss**: Number of missed Trypsin cleavage sites. **Ions score**: The Ions Score is calculated by the Mascot search engine for each peptide matched from MS/MS peak lists. This score is based on the probability that ion fragmentation matches are non-random events. If the Ion Score is equal to or greater than the Mascot® Significance Level calculated for the database search, the peptide match is considered to be statistically non-random at the 95% confidence interval. Ions score = -10*Log(*P*), where *P* is the probability that the observed match is a random event. **Best Ions score:** The highest individual Ion Score for a given protein identification. **Expect**: Ions score expectation value. **Peptide:** The amino acid sequence of the selected peptide. **Variable Modification**: Variable modification type on the peptide.

New Phytologist

	Identified protein			PMF data					MSMS data												
Id nr	Locus	Name	# Found/ # exp	Protein Score	Expect	RMS error (ppm)	Sequence coverage %	Unique Peptides	Total Ion Score	Peptide Number	Start	End	Observed	Mr (Exp)	Mr (Calc)	Delta (Da)	Miss	Ions Score	Expect	Peptide	Variable Modification
1	AT3G05680	VIR	2/2	1150	3.50E-111	4	37	50	661	15	123	134	1391	1390	1390	-0.003	0	91	2.10E-08	K.DLGQYNIILEGR.S	
1										21	1816	1831	1663	1662	1662	0.002	0	28	2.90E-02	K.SGQQHTGHIHGGFSGR.G	
1										23	371	384	1688	1687	1687	-0.006	0	35	5.00E-03	R.HSVGCEGFLGWWPR.E	
1										27	1166	1180	1753	1752	1752	-0.006	0	75	4.90E-07	R.YLNFLASLLEHPHAK.G	
1										31	1461	1477	1821	1820	1820	0.010	0	31	1.90E-02	K.GVFVSPGNLDMDDLVSR.N	
1										45	1103	1120	2043	2042	2042	-0.003	0	46	1.50E-04	K.IIQHHAVSALVVIQDMLR.V	
1										51	619	636	2103	2102	2102	-0.002	0	29	2.80E-02	K.GFTCSLLEIGINLEMHLR.V	
1										54	837	856	2125	2124	2124	-0.001	0	121	1.50E-11	K.SFEGVNLSDSSISQLTTALR.I	
1										56	1487	1503	2192	2191	2191	-0.004	1	37	2.30E-03	R.GLEDKFWWECPETLPER.L	
1										62	576	596	2319	2318	2318	-0.006	0	48	6.90E-05	R.TGLSFLLHHPELTATIIQSLK.G	
1										65	1210	1230	2395	2394	2394	-0.008	0	45	4.20E-04	R.VLEYGIVSASSVIQWCIPAFR.S	
1										70	1885	1907	2512	2511	2511	0.003	0	0	1.30E+01	R.LMPPLPSAIPQYSSNPYASLPPR.T	Oxidation (M)
1										74	135	159	2694	2693	2693	0.004	0	34	5.10E-03	R.SVTDIVSSTEGNLEDLPLVLHSVNR.T	
1										80	79	106	3199	3198	3198	-0.001	0	39	9.00E-04	R.LCNPFLYTPSAPYPLEVEAVVTNHLVVR.G	
2	AT5G01160	HAKAI	2/2	237	7.10E-20	3	24	7	189	9	75	84	1212	1211	1211	0.006	0	70	2.30E-06	R.CDFPIAIYGR.L	
2										18	343	354	1421	1420	1420	-0.001	0	43	4.90E-04	R.DGQSFGWPQENR.D	
2										25	44	59	1582	1581	1581	-0.004	0	45	1.40E-04	K.GIGSVTPTTVIKPVGR.R	
2										31	85	98	1762	1761	1761	-0.003	0	31	7.30E-03	R.LIPCDHAFCLECAR.S	
3	AT3G54170	FIP37	2/2	229	4.50E-19	5	33	9	175	16	105	114	1267	1266	1266	-0.009	0	67	3.70E-06	R.FLIDYIQNLK.S	
3										34	169	181	1540	1539	1539	-0.002	0	50	2.00E-04	R.LLLDPAIHEEFSR.L	
3										43	168	181	1696	1695	1695	-0.011	1	19	2.00E-01	R.RLLLDPAIHEEFSR.L	
3										67	28	50	2442	2441	2441	0.005	0	39	1.20E-03	R.SFGDLEDDEDDIFGSTTVAPGVR.T	
4	AT4G09980	MTB	1/2	88.9	4.60E-05	12	13	9	50	11	786	794	1056	1055	1055	0.002	0	38	2.30E-03	R.IIEHFALGR.R	
4										21	796	807	1499	1498	1498	0.000	1	12	1.30E+00	R.RLELFGEDHNIR.A	
